# Development and Internal Validation of an Interpretable Machine Learning Model for Identifying Past-Year Nonsuicidal Self-Injury Among Adolescents With Depression: Retrospective Study

**DOI:** 10.2196/95340

**Published:** 2026-10-08

**Authors:** Gaoyang Liu, Chenxi Yang, Wendan Chen, Ningning Ding, Wenwen Tian, Xinwu Ye, Mengdan Luo, Xinnan Mao, Guohua Zhang, Ke Zheng

**Affiliations:** 1 School of Nursing Anqing Medical College Anqing, Anhui China; 2 School of Mental Health Wenzhou Medical University Wenzhou, Zhejiang China; 3 School of Technology and Data Yantai Nanshan University Yantai, Shandong China; 4 Wenzhou Seventh People’s Hospital Wenzhou, Zhejiang China; 5 School of Nursing Wenzhou Medical University Wenzhou, Zhejiang China; 6 Zhejiang Provincial Clinical Research Center for Mental Disorders Kangning Hospital of Wenzhou Medical University Wenzhou, Zhejiang China; 7 Key Research Center of Philosophy and Social Sciences of Zhejiang Province (Institute of Medical Humanities) Wenzhou Medical University Wenzhou, Zhejiang China

**Keywords:** nonsuicidal self-injury, depressive disorders, adolescents, machine learning, biopsychosocial approach

## Abstract

**Background:**

Nonsuicidal self-injury (NSSI) is common among adolescents with depressive disorders and is associated with substantial clinical burden. Machine learning may assist in identifying multidimensional patterns associated with NSSI, although its incremental value beyond direct clinical assessment remains uncertain.

**Objective:**

This study compares 7 supervised machine learning model variants for classifying past-year NSSI status among adolescents with depressive disorders and conducts detailed analyses of interpretability, sensitivity, subgroup performance, and clinical utility using a random forest model as the focal exploratory model.

**Methods:**

We retrospectively identified 437 adolescents aged 10-19 years who received inpatient or outpatient psychiatric care at 4 hospitals in Zhejiang Province, China, between January 2021 and December 2023. A total of 410 eligible participants were included, of whom 274 reported NSSI during the preceding year. The primary analysis included 67 assessment-time sociodemographic, physiological, biochemical, and clinical-behavioral features. The sample was divided into a training set (287/410, 70%) and an untouched held-out test set (123/410, 30%) using stratified random sampling. Hyperparameters were optimized using 5-fold cross-validation within the training set. Performance was evaluated using discrimination, classification metrics, calibration, and decision-curve analysis. Random forest model behavior was examined using Gini importance, permutation importance, and Shapley Additive Explanations (SHAP).

**Results:**

The radial basis function support vector machine achieved the highest mean cross-validated area under the receiver operating characteristic curve (AUROC) in the training set (0.720), whereas the random forest showed the highest observed AUROC and area under the precision-recall curve (AUPRC) and the lowest Brier score in the held-out test set. The random forest achieved an AUROC of 0.681 (95% CI 0.583-0.778), an AUPRC of 0.808 (95% CI 0.741-0.874), an accuracy of 0.667 (95% CI 0.585-0.748), a sensitivity of 0.720 (95% CI 0.622-0.817), a specificity of 0.561 (95% CI 0.415-0.707), and a Brier score of 0.212 (95% CI 0.188-0.236). Its calibration slope was 1.013. Pairwise AUROC differences between the random forest and alternative models were not statistically significant after Holm correction (Holm-adjusted *P* values ranged from .17 to .81). The full random forest model did not outperform a suicidal-ideation-only model (AUROC 0.701), whereas excluding suicidal ideation and previous suicide attempt reduced the AUROC to 0.540. A biochemical-only model showed near-chance discrimination (AUROC 0.513). Decision-curve analysis indicated that the random forest provided greater net benefit than the assess-all and assess-none strategies across threshold probabilities of approximately 0.51-0.80. Suicidal ideation showed the most consistent importance across Gini, SHAP, and permutation analyses.

**Conclusions:**

The random forest had the highest observed AUROC and AUPRC and the lowest Brier score in the held-out test set among the evaluated algorithms, although the pairwise AUROC differences were not statistically significant after correction for multiple comparisons. Suicidality-related information contributed substantially to model discrimination, whereas routinely collected biochemical variables did not demonstrate clear incremental value. Because this was a retrospective cross-sectional classification study, the findings do not establish prospective risk prediction. External and prospective temporal validation is required before clinical implementation.

## Introduction

Depressive disorders are among the most common mental health conditions during adolescence and are associated with substantial impairments in emotional well-being, academic functioning, interpersonal relationships, and daily life [1]. Adolescents with depressive disorders are also particularly vulnerable to nonsuicidal self-injury (NSSI), defined as the deliberate and direct injury of body tissue without suicidal intent [2]. Meta-analytic and epidemiological studies suggest that NSSI affects a substantial proportion of adolescents, with prevalence estimates generally higher in psychiatric and depressive-disorder samples than in community populations [3-6]. Although NSSI is conceptually distinguished from suicidal behavior by the absence of an intention to die, it is associated with greater psychiatric severity, repeated self-harm, suicidal thoughts and behaviors, and increased demand for mental health care [7-9]. Consequently, accurate identification and comprehensive assessment of recent NSSI among adolescents with depressive disorders represent important clinical priorities.

The clinical assessment of NSSI remains challenging because self-injurious behavior is heterogeneous and may vary in frequency, methods, functions, medical severity, and persistence [6,10,11]. Adolescents may also conceal NSSI because of shame, fear of stigma, concerns about confidentiality, or uncertainty about how parents and clinicians will respond [12,13]. Direct inquiry regarding NSSI and suicidal thoughts therefore remains essential. However, multidimensional clinical information may help clinicians identify patterns associated with recent NSSI and determine which patients require a more comprehensive, structured assessment [2,12]. Importantly, models based on concurrently or retrospectively measured variables can classify current or previous NSSI status, but they cannot establish temporal precedence or predict future NSSI onset unless they are evaluated prospectively using longitudinal data [14-18].

NSSI is understood as a complex behavior arising from interactions among psychological distress, behavioral vulnerability, interpersonal experiences, family context, and biological or physiological characteristics [19,20]. Established clinical correlates include younger age, female sex, greater depressive severity, emotional dysregulation, suicidal ideation, previous suicide attempts, and other forms of self-directed or interpersonal aggression [6,9,21,22]. Family-level factors are also particularly relevant during adolescence. Parental psychopathology, family conflict, adverse childhood experiences, limited perceived parental support, maladaptive parenting practices, and difficulties in parent-adolescent communication have all been associated with adolescent NSSI [23-25]. These factors may operate as vulnerabilities, protective resources, or moderators of the relationship between emotional distress and self-injurious behavior.

Physiological and biochemical characteristics have also been explored in relation to depression and self-injurious behavior, including inflammatory markers, thyroid function, metabolic indicators, liver and renal function, and neuroendocrine measures [26,27]. Such routinely collected variables may contain information that is not fully captured by symptom-based assessments and may contribute to nonlinear or interactive model structures [28]. Nevertheless, evidence that individual laboratory markers provide clinically meaningful information beyond established clinical variables remains inconsistent [18]. Laboratory measurements may reflect current depressive severity, medication exposure, nutritional status, physical activity, comorbid conditions, or consequences of recent behavior rather than antecedent mechanisms of NSSI. Accordingly, biochemical variables identified by a cross-sectional model should be regarded as potential model correlates and hypothesis-generating findings rather than independent causal biomarkers [18,19].

Despite the relevance of family context, family-level information is frequently underrepresented in routinely collected electronic medical records. Variables such as family psychiatric history or household residence may be available, whereas standardized assessments of parental mental health, family conflict, parenting practices, perceived support, parent-adolescent communication, and adverse childhood experiences are often absent. This limitation is particularly important in pediatric settings, where parents or caregivers are not only sources of clinical information but also potential partners in safety planning, treatment engagement, and relapse prevention [29]. At the same time, communicating model-derived findings to parents requires careful attention to adolescent autonomy, confidentiality, uncertainty, and the risk of stigmatizing labels [12,30]. Parent-facing information should therefore be accessible, probabilistic, and nondeterministic, and should be communicated within a clinician-mediated assessment rather than through automated alerts or isolated risk scores.

Machine learning has increasingly been applied to the study of NSSI and related self-harm outcomes using questionnaire data, clinical records, behavioral information, digital phenotyping, neuroimaging, and biological measures [31-34]. Compared with conventional regression approaches, some machine learning algorithms can accommodate complex nonlinear relationships and higher-order interactions [28,35]. Random forests are particularly suitable for modeling heterogeneous clinical data and allow complementary examination through impurity-based importance, permutation importance, and Shapley Additive Explanations (SHAP) [36]. However, algorithmic complexity does not necessarily ensure clinical usefulness. Most published NSSI models have been internally evaluated in cross-sectional or geographically restricted samples, whereas fewer studies have examined future NSSI occurrence using longitudinal designs [17,18,31-33]. Additional limitations include small event-to-feature ratios, inadequate control of information leakage, insufficient reporting of hyperparameter optimization and missing-data procedures, limited assessment of calibration, absence of external validation, and failure to compare complex models with simple clinical screens [30,35].

Model interpretability is particularly important when machine learning is applied in mental health care [30]. SHAP can quantify the contribution of a feature to an individual model output and summarize the magnitude and direction of contributions across a sample [36]. Nevertheless, SHA*P* values explain the predictions of a fitted model rather than the underlying causal process. A high SHAP or Gini ranking does not demonstrate that a feature is independently associated with NSSI, nor does it establish that changing the feature would alter clinical outcomes. Interpretation should therefore be supported by calibration, sensitivity analyses, subgroup performance, comparisons with simpler models, and alternative importance measures such as test-set permutation importance [30,36].

Accordingly, this retrospective multicenter cross-sectional study aimed to compare 7 supervised machine learning model variants for classifying past-year NSSI status among adolescents with depressive disorders using routinely collected sociodemographic, physiological-biochemical, and clinical-behavioral information. We evaluated discrimination, classification performance, calibration, and clinical net benefit in an untouched, held-out test set. As suicidal ideation is both a strong clinical correlate of NSSI and an immediate assessment priority, we examined whether the multidimensional models provided incremental information beyond suicidal ideation and other basic clinical variables. We also evaluated performance after excluding suicidality-related variables and examined sex- and residence-specific performance. The random forest was used as the focal exploratory model for detailed analyses of Gini, permutation importance, and SHAP. Finally, we considered the limited availability of family-context variables and the implications of model-assisted assessment for future parent- and adolescent-involved clinical communication. Given the concurrent and retrospective measurement of the candidate features and outcome, this study was designed to evaluate internal classification rather than prospective prediction of future NSSI.

## Methods

### Study Design and Participants

This retrospective multicenter cross-sectional study screened the electronic medical records of adolescents aged 10-19 years with depressive disorders who received inpatient or outpatient psychiatric care at 4 hospitals in Zhejiang Province, China, between January 2021 and December 2023. The study was designed to classify past-year NSSI status rather than to predict the future onset or recurrence of NSSI. Participants were retrospectively identified through systematic screening of electronic medical records and screened for eligibility. The inclusion criteria were as follows: (1) age between 10 and 19 years; (2) a clinical diagnosis of depressive disorder according to the International Classification of Diseases, 10th Revision (ICD-10); and (3) availability of the clinical assessment and laboratory information required for this analysis. The exclusion criteria were as follows: (1) bipolar disorder, schizophrenia-spectrum disorder, or another severe psychiatric disorder; (2) intellectual disability or cognitive impairment that could prevent a reliable clinical assessment; (3) severe or unstable physical illness or a chronic medical condition likely to substantially affect physiological or biochemical measurements; and (4) substance use disorder.

After the initial screening of the electronic medical records, 2 senior psychiatrists independently evaluated participant eligibility. Disagreements were reviewed with a third psychiatrist and resolved through discussion. A total of 437 records were screened, of which 27 were excluded because of severe psychiatric disorders (n=12), intellectual disability or cognitive impairment (n=2), severe or unstable physical illness (n=5), or substance use disorder (n=8). The remaining 410 participants were included in the final analytic sample. The participant-selection and modeling procedures are presented in Figure 1. Missing data within the included dataset were addressed using multiple imputation.

**Figure 1:**
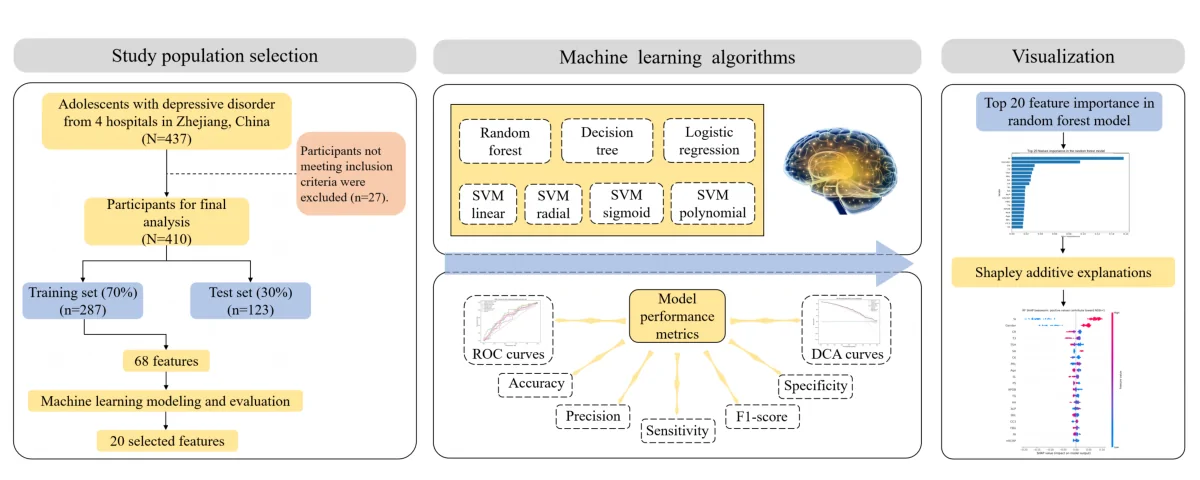
Study flowchart. AA: amylase; ALP: alkaline phosphatase; APOB: apolipoprotein B; CC3: complement component C3; CK: creatine kinase; CR: creatinine; DCA: decision curve analysis; FA: free fatty acid; FBG: fasting blood glucose; HSCRP: high-sensitivity C-reactive protein; IBIL: indirect bilirubin; PRL: prolactin; PS: previous suicide attempt; RF: random forest; ROC: receiver operating characteristic; SA: suicide attempt; SHAP: Shapley Additive Explanations; SI: self-injury; SL: aspartate aminotransferase-to-alanine aminotransferase ratio; SVM: support vector machine; T3: total triiodothyronine; T4: total thyroxine; TG: triglyceride; TSH: thyroid-stimulating hormone.

### Ethics Approval

The Ethics Committee of Kangning Hospital, affiliated with Wenzhou Medical University, approved the study (ethical approval number KNLL-20211011002). The study was performed in strict accordance with the ethical principles of the Declaration of Helsinki (1975).

### Data Collection

#### Outcome Variable

Past-year NSSI status was assessed during clinical interviews conducted independently by 2 senior psychiatrists. Participants were asked, “During the past year, have you intentionally injured yourself without intending to die, for example, by cutting, scratching, burning your skin, or hitting yourself?” Responses were recorded as “yes” or “no,” with NSSI coded as 1 and non-NSSI coded as 0. Any disagreement between the 2 psychiatrists was reviewed by a third psychiatrist and resolved by consensus.

The binary outcome represented the presence or absence of broadly defined NSSI during the preceding 12 months. It did not constitute a diagnosis of NSSI disorder or capture NSSI frequency, persistence, methods, medical severity, functions, or associated impairment.

#### Candidate Features

##### Overview

Candidate features were identified through expert consultation and a review of the relevant literature. The processed dataset contained 71 candidate feature columns. Three fields that were not consistently defined in the original manuscript or source coding—SS, number of hospitalizations, and creatine kinase muscle-brain isoenzyme—were excluded from the manuscript-matched feature set. Completed length of stay was excluded from the primary model because it was not available at the index assessment and could have been affected by subsequent clinical management. The primary assessment-time model therefore contained 67 candidate features, grouped into sociodemographic and lifestyle features, family and social-context features, physiological and biochemical features, and clinical and behavioral features. The complete feature list is provided in Table S1 in Multimedia Appendix 1.

##### Sociodemographic and Lifestyle Features

Sociodemographic and lifestyle information was systematically collected during routine psychiatric consultations. The variables included sex, age, education level, BMI (kg/m^2^), place of residence, smoking status, drinking status, family psychiatric history, and religious belief.

##### Family and Social-Context Features

The family and contextual variables available in the routinely collected electronic medical records were family psychiatric history and place of residence. Family psychiatric history indicated whether a psychiatric disorder had been documented among family members. Place of residence was classified according to the urban-rural coding used in the source records and was considered a contextual indicator rather than a direct measure of family functioning.

Standardized assessments of parental psychopathology, family conflict, perceived parental support, parent-adolescent communication, parenting style, and adverse childhood experiences were not systematically collected across the 4 participating hospitals and were therefore unavailable for model development.

##### Physiological and Biochemical Features

Blood samples were collected from the antecubital vein by trained nursing staff after overnight fasting (≥8 hours) between 7 and 9 AM upon hospital admission, ensuring standardized preanalytical conditions. Blood coagulation function was assessed by measuring D-dimer using a latex-enhanced immunoturbidimetric assay (Sysmex CS-5100). Thyroid function was evaluated by measuring thyroid peroxidase antibodies, thyroid-stimulating hormone (TSH), free triiodothyronine, free thyroxine, total triiodothyronine (T3), and total thyroxine (T4) using a chemiluminescence immunoassay (Siemens ADVIA Centaur XP). Liver function was assessed by measuring alanine aminotransferase, aspartate aminotransferase, the aspartate aminotransferase-to-alanine aminotransferase ratio, total protein, albumin, globulin, albumin-to-globulin ratio, alkaline phosphatase, gamma-glutamyl transferase, total bilirubin, total bile acid, direct bilirubin, and indirect bilirubin using automated enzymatic methods (cobas c702; Roche). Serum prealbumin was determined using an immunonephelometric assay (Siemens BN ProSpec), while alpha-L-fucosidase was quantified using a spectrophotometric assay (Hitachi 7600). Markers of glucose and lipid metabolism, including fasting blood glucose, triglycerides, total cholesterol, high-density lipoprotein, low-density lipoprotein, apolipoprotein A, apolipoprotein B, lipoprotein a, and free fatty acids, were assessed using an enzymatic colorimetric assay (cobas c702).

Renal function was evaluated by measuring blood urea nitrogen, creatinine, and uric acid using an enzymatic method (cobas c702). Myocardial and muscle enzyme activities, including creatine kinase, creatine kinase isoenzymes (muscle-brain isoenzyme), and lactate dehydrogenase, were analyzed using an enzymatic assay (cobas c702). Plasma homocysteine levels were measured using an enzymatic cycling assay (cobas c702). Electrolyte balance was assessed by measuring potassium, sodium, chloride, calcium, magnesium, and phosphorus levels using an ion-selective electrode method (cobas c702).

Biochemical markers included serum ferritin, measured using an immunoturbidimetric assay (cobas c702), and cholinesterase, assessed using a butyrylthiocholine substrate method (cobas c702). Inflammatory response was evaluated by measuring high-sensitivity C-reactive protein using a latex-enhanced immunoturbidimetric assay (cobas c702). Complement system activity was assessed by measuring complement component C3 and complement component C4 using an immunonephelometric assay (Siemens BN ProSpec). Lastly, amylase was analyzed using an enzymatic assay (cobas c702), and the prolactin level was determined using a chemiluminescent immunometric assay (Siemens ADVIA Centaur XP).

##### Clinical and Behavioral Features

Data on length of hospital stay (days), disease course (months), and first episode were obtained from electronic medical records. The depression grade and psychotic symptoms were systematically assessed through structured clinical interviews conducted by trained psychiatrists. The depression grade was classified as mild, moderate, or severe according to ICD-10 criteria based on clinical assessments of symptom intensity, functional impairment, and distress levels. Psychotic symptoms were evaluated based on DSM-5 (Diagnostic and Statistical Manual of Mental Disorders, Fifth Edition) diagnostic criteria, with patients systematically assessed for depressive hallucinations and depressive delusions, and symptoms recorded as either present or absent. Suicidal ideation was assessed according to DSM-5 criteria, with patients asked, “In the past month, have you seriously considered ending your life?” Suicide attempt history was assessed using the question, “Have you ever made an actual suicide attempt in your lifetime (eg, overdosing or cutting your wrists with suicidal intent)?” Participants were asked to respond “Yes” or “No.” Aggressive behaviors were documented through both direct clinical observation and structured interviews with caregivers and patients. Verbal aggression was assessed using a single question: “Has the patient recently exhibited hostile, insulting, or aggressive language toward others?” Physical aggression was assessed using the question: “Has the patient exhibited any aggressive physical behavior (eg, hitting, pushing, or fighting) in the past month?” Participants were asked to respond “Yes” or “No.”

### Patient and Parent Involvement

Adolescents and parents or caregivers were not involved in the original study design, candidate-feature selection, model development, interpretation of the findings, or preparation of the manuscript. Written informed consent was obtained from participants and their legal guardians as part of the ethical procedures but did not constitute patient or parent involvement in research co-design.

Future prospective implementation studies should involve adolescents, parents or caregivers, and clinicians in determining how model outputs, uncertainty, privacy, and the distinction between NSSI and suicidal intent should be communicated. Any parent-facing use of model-derived information should occur within a clinician-mediated assessment rather than through an automated alert or an isolated risk score.

### Statistical Analysis

#### Descriptive Analysis and Data-Quality Assessment

The revised statistical and machine learning analyses were conducted in Python (Python Foundation) using pandas, NumPy, SciPy, statsmodels, scikit-learn, imbalanced-learn, and SHAP. All statistical tests were 2-sided, and a *P* value of <.05 was considered statistically significant.

Continuous variables were summarized using medians and IQRs and compared between the NSSI and non-NSSI groups using 2-sided Mann-Whitney *U* tests. Categorical variables were summarized using counts and percentages. Pearson chi-square tests were used for categorical comparisons, except for 2×2 tables with expected cell counts below 5, for which Fisher exact tests were used. These univariable comparisons were descriptive and were not used to screen or select features for model development.

Automated range checks and category-level audits were conducted before model fitting. Potentially implausible numerical values and unexpected category codes were flagged for verification against the source records. No values were corrected automatically. Any correction was required to be supported by the source record and documented in an auditable correction file before the complete analysis was rerun.

#### Sample-Size Considerations

All eligible records available during the predefined study period were included; therefore, no prospective sample-size calculation was performed. The primary model contained 67 candidate variables and generated 88 model inputs after one-hot encoding. The analytic sample included 274 participants with NSSI and 136 without NSSI, corresponding to 4.09 NSSI cases and 2.03 non-NSSI cases per candidate variable. After one-hot encoding, there were 3.11 NSSI cases and 1.55 non-NSSI cases per model input. These ratios indicated a substantial risk of overfitting and model-selection instability; therefore, the modeling analyses were considered exploratory.

#### Model Development

The 410 participants were divided by stratified random sampling into a training set (287/410, 70%) and a held-out test set (123/410, 30%), using a random seed of 46. The training set contained 287 participants, including 192 with NSSI and 95 without NSSI. The held-out test set contained 123 participants, including 82 with NSSI and 41 without NSSI. The test set was not used for preprocessing estimation, hyperparameter tuning, resampling, or model fitting. The synthetic minority over-sampling technique was applied to address class imbalance and enhance the representation of the minority class [37].

Categorical features were imputed within the modeling pipeline using the most frequent category and subsequently one-hot encoded. Continuous variables were imputed using the median within the pipeline. Continuous features were standardized for logistic regression and support vector machine models using parameters estimated only from the corresponding training data or cross-validation fold. Continuous features were not standardized for decision tree or random forest models. All preprocessing procedures were incorporated into the model pipelines to prevent information leakage.

Seven prespecified supervised machine learning model variants were evaluated: logistic regression, decision tree, random forest, and support vector machines with linear, radial basis function, polynomial, and sigmoid kernels. Class weights were balanced in the primary analysis to address the unequal frequencies of NSSI and non-NSSI. Synthetic oversampling was not applied before the training-test split or to the held-out test set.

Hyperparameters were optimized using randomized search with stratified 5-fold cross-validation exclusively within the training set. Area under the receiver operating characteristic curve (AUROC) was used as the optimization metric. Up to 30 hyperparameter configurations were sampled for each model; when a search space contained fewer than 30 possible combinations, all possible combinations were evaluated.

The selected random forest configuration used 1500 trees, a maximum tree depth of 15, a minimum of 5 observations required to split an internal node, a minimum of 12 observations per terminal leaf, 44 of the 88 (50%) available model inputs considered at each split, the Gini impurity criterion, bootstrap sampling, and balanced class weights.

The SVM with a radial basis function kernel achieved the highest mean AUROC during training-set cross-validation. After all 7 optimized models were evaluated on the held-out test set, the random forest showed the highest observed test-set AUROC and area under the precision-recall curve (AUPRC) and the lowest observed Brier score. The random forest was therefore designated post hoc as the focal exploratory model for detailed calibration, decision-curve, subgroup, sensitivity, and interpretability analyses. This focal designation did not imply statistically significant superiority over the alternative models.

#### Model Performance, Calibration, and Formal Comparison

Model discrimination was evaluated in the held-out test set using the area under the receiver operating characteristic curve and the area under the precision-recall curve. At the prespecified probability threshold of 0.50, accuracy, sensitivity, specificity, positive predictive value, negative predictive value, and *F*_1_-score were calculated. NSSI was defined as the positive class.

The 95% CIs for AUROC, AUPRC, accuracy, sensitivity, specificity, positive predictive value, negative predictive value, *F*_1_-score, and Brier score were estimated using 2000 stratified bootstrap resamples of the held-out test set.

Calibration was evaluated using the Brier score, calibration intercept, calibration slope, and calibration plots based on quantile-grouped predicted probabilities. Ideal calibration was represented by a calibration intercept of 0 and a calibration slope of 1.

The random forest was compared with each of the other 6 model variants using paired DeLong tests based on predictions from the same held-out test participants. Holm correction was applied to account for multiple pairwise comparisons. Model rankings based solely on observed test-set AUROCs were not interpreted as evidence of superiority when the adjusted comparisons were not statistically significant.

Clinical utility was assessed through decision curve analysis to quantify the net clinical benefit of model-based decision-making relative to other strategies (eg, treating all or treating none) across a range of threshold probabilities [38]. Feature importance was assessed using mean decrease in Gini impurity, an impurity-based measure commonly used to quantify variable importance in random forest models [39]. Additionally, SHAP was used to enhance model interpretability by quantifying the contribution of individual features to model predictions [36]. The SHAP approach offers both global and individual-level explanations, improving the transparency of the model and facilitating interpretation in clinical applications.

Decision curve analysis was performed over threshold probabilities from 0.01 to 0.80 to quantify net benefit relative to strategies of assessing all participants and assessing no participants. The modeled clinical action was referral for a more comprehensive, structured assessment of NSSI rather than automatic diagnosis, hospitalization, or treatment.

#### RF-Focused Incremental-Value and Sensitivity Analyses

A series of RF-focused analyses was conducted to examine the incremental contribution of different feature domains and the potential dominance of suicidality-related variables. These analyses included an RF model using suicidal ideation alone; an RF model using suicidal ideation and previous suicide attempt; a basic clinical RF model; a clinical-only RF model; a biochemical-only RF model; an RF model using only the available family and contextual variables; a full RF model excluding suicidal ideation and previous suicide attempt; a full RF model excluding sex; a full RF model excluding residence; a full RF model excluding family psychiatric history and residence; and a full RF model including completed length of stay.

Logistic regression models using suicidal ideation alone and suicidal ideation together with previous suicide attempt were additionally fitted as simple clinical benchmarks. For the RF ablation analyses, the RF hyperparameters were fixed at the values selected for the full RF model and were not retuned separately for each reduced feature set.

The full RF model was formally compared with the reduced and benchmark models using paired DeLong tests, with Holm correction for multiple comparisons. These analyses were used to determine whether the multidimensional RF model provided incremental discrimination beyond the direct assessment of suicidal ideation and other basic clinical information.

As a class-imbalance sensitivity analysis, the RF was refitted using the Synthetic Minority Over-Sampling Technique for Nominal and Continuous (SMOTENC). SMOTENC was applied exclusively to the training data, with 5 nearest neighbors, and was never applied to the held-out test set. The RF hyperparameters were fixed at those selected in the primary training-set search.

#### Subgroup Analyses

RF performance was evaluated separately within the sex and residence subgroups represented in the held-out test set. Within each subgroup, AUROC, AUPRC, accuracy, sensitivity, specificity, positive predictive value, negative predictive value, *F*_1_-score, Brier score, and corresponding bootstrap CIs were calculated when both outcome classes were present.

The subgroup analyses were exploratory and were not used to select separate subgroup-specific models or classification thresholds. Hospital-specific performance and leave-one-hospital-out validation could not be evaluated because the processed analytic dataset did not contain a hospital-site identifier.

#### RF Model Interpretation

The focal RF model was interpreted using 3 complementary approaches: Gini impurity–based feature importance, test-set permutation importance, and SHAP. Gini importance was aggregated from one-hot-encoded inputs to the corresponding original variables.

Permutation importance was calculated in the held-out test set using AUROC as the scoring measure and 100 repeated permutations per feature. A positive value represented a reduction in test-set AUROC after a feature was permuted, whereas a value near or below 0 indicated a limited or unstable contribution to held-out discrimination.

SHA*P* values were calculated using TreeExplainer and observations from the held-out test set. SHA*P* values were aggregated from encoded inputs to their original variables. Mean absolute SHA*P* values were used to summarize feature importance magnitude. Beeswarm plots were used to display the direction and distribution of feature contributions, with positive SHA*P* values indicating contributions toward the NSSI class. Dependence plots were generated for high-ranking continuous features to examine possible nonlinear and threshold-like patterns.

The agreement between Gini and mean absolute SHAP rankings was assessed using Spearman rank correlation. Gini, SHAP, and permutation importance were compared to identify variables with relatively consistent importance across methods. Model-specific feature importance and SHA*P* values were interpreted as descriptions of fitted-model behavior and not as evidence of independent association, causality, temporal precedence, or treatment targets.

All 67 candidate features remained in the full RF model. The 20 variables displayed in the Gini and SHAP figures represented only the 20 highest-ranked variables for visual clarity and did not constitute a separately selected 20-feature model.

## Results

### Participant Characteristics

A total of 437 electronic medical records were screened. After application of the eligibility criteria, 27 records were excluded, leaving 410 adolescents with depressive disorders in the final analytic sample. Of these participants, 274 (66.8%) reported NSSI during the preceding 12 months and 136 (33.2%) did not. Stratified random sampling allocated 287 participants to the training set, including 192 with NSSI and 95 without NSSI, and 123 participants to the untouched held-out test set, including 82 with NSSI and 41 without NSSI.

The processed analytic dataset contained no missing values, and all 410 participants were included in the complete-case analysis. The primary assessment-time model contained 67 candidate variables and generated 88 model inputs after one-hot encoding. This corresponded to 4.09 NSSI cases and 2.03 non-NSSI cases per candidate variable, and 3.11 NSSI cases and 1.55 non-NSSI cases per encoded model input.

The median age of the overall sample was 15 (IQR 13-16) years, and 320 of the 410 (78.1%) participants were female. Participants with NSSI were younger than those without NSSI (median 14 years, IQR 13-16 years vs median 15 years, IQR 14-16 years; *P*<.001). The proportion of female participants was also higher in the NSSI group than in the non-NSSI group (232/274, 84.7% vs 88/136, 64.7%; *P*<.001). Education-level distribution differed between the groups (*P*=.008). Smoking was uncommon but was reported by 11 participants in the NSSI group and none in the non-NSSI group (11/274, 4% vs 0/136, 0%; *P*=.02). No statistically significant between-group differences were observed for BMI (*P*=.72), place of residence (*P*=.36), drinking status (*P*=.06), family psychiatric history (*P*=.84), or religious belief (*P*=.21). Other sociodemographic characteristics are presented in Table 1.

**Table 1:** Comparison of characteristics of depressed adolescents between the NSSI^a^ group and the non-NSSI group (n=410).

Sociodemographic variables			Total sample		NSSI				*P* value
					No (n=136)		Yes (n=274)		
**Gender, n (%)**									*<.001* ^b^
	Male	90 (22.0)		48 (53.3)		42 (46.7)			
	Female	320 (78.0)		88 (27.5)		232 (72.5)			
Age (years), median (IQR)			15 (13-16)		15 (14-16)		14 (13-16)		*<.001*
**Education level, n (%)**									*.006*
	Primary school or below	27 (6.6)		4 (14.8)		23 (85.2)			
	Junior middle school	232 (56.6)		69 (29.7)		163 (70.3)			
	Senior high school	151 (36.8)		63 (41.7)		88 (58.3)			
BMI (kg/m^2^), median (IQR)			20.37 (18.49-22.85)		20.34 (18.74-22.86)		20.38 (18.41-22.83)		.72
**Residence, n (%)**									.36
	Urban	228 (55.6)		80 (35.1)		148 (64.9)			
	Rural	182 (44.4)		56 (30.8)		126 (69.2)			
**Smoking, n (%)**									*.02*
	Yes	11 (2.7)		0 (0.0)		11 (100.0)			
	No	399 (97.3)		136 (34.1)		263 (65.9)			
**Drinking, n (%)**									.06
	Yes	7 (1.7)		0 (0.0)		7 (100.0)			
	No	403 (98.3)		136 (33.7)		267 (66.3)			
**Family psychiatric history, n (%)**									.84
	Yes	14 (3.4)		5 (35.7)		9 (64.3)			
	No	396 (96.6)		131 (33.1)		265 (66.9)			
**Religious belief, n (%)**									.21
	Yes	35 (8.5)		15 (42.9)		20 (57.1)			
	No	375 (91.5)		121 (32.3)		254 (67.7)			

^a^NSSI: nonsuicidal self-injury.

^b^Italicized values are statistically significant (*P*<.05).

Physiological and biochemical characteristics are presented in Table 2. Among the continuous physiological and biochemical variables, participants with NSSI had lower creatinine concentrations than those without NSSI (median 59 μmol/L, IQR 52-65 μmol/L vs median 63 μmol/L, IQR 54-72 μmol/L; *P*=.001), lower creatine kinase concentrations (median 64.5 U/L, IQR 51-82 U/L vs median 76 U/L, IQR 55-94.8 U/L; *P*=.005), and lower fructosamine concentrations (median 1.97 mmol/L, IQR 1.86-2.11 mmol/L vs median 2.04 mmol/L, IQR 1.92-2.17 mmol/L; *P*=.009). No other continuous variable showed a statistically significant between-group difference. In particular, high-sensitivity C-reactive protein did not differ significantly between the NSSI and non-NSSI groups (*P*=.19). These univariable comparisons were descriptive and were not used for feature selection or model development.

Clinical and behavioral characteristics according to past-year NSSI status are summarized in Table 3. Suicidal ideation and previous suicide attempt showed different distributions between participants with and without past-year NSSI. Suicidal ideation was reported by 221 of 274 (80.7%) participants with NSSI and 66 of 136 (48.5%) participants without NSSI (*P*<.001). A previous suicide attempt was reported by 88 (32.1%) participants with NSSI and 20 (14.7%) participants without NSSI (*P*<.001). The distribution of psychotic-symptom categories differed between the groups (*P*=.04), whereas depression-severity categories did not (*P*=.52). First-episode status (*P*=.11), illness duration (*P*=.08), verbal aggression (*P*=.26), and physical aggression (*P*=.62) also did not differ significantly between the groups.

**Table 2:** Physiological and biochemical characteristics according to past-year NSSI^a^ status.

Physiological and biochemical variables	Total sample, median (IQR)	NSSI		*P* value
		No (n=136), median (IQR)	Yes (n=274), median (IQR)	
D-dimer (µg/mL)	0.22 (0.16-0.3)	0.22 (0.16-0.283)	0.22 (0.16-0.31)	.86
Thyroid peroxidase antibody (kU/L)	11 (8.47-16)	11.4 (8.54-16.2)	11 (8.41-15.8)	.51
Thyroid-stimulating hormone (µIU/mL)	1.46 (1.02-2.1)	1.52 (1.04-2.21)	1.43 (1.02-2.08)	.49
Free triiodothyronine (pmol/L)	5.19 (4.67-5.64)	5.21 (4.68-5.63)	5.16 (4.66-5.66)	.96
Free thyroxine (pmol/L)	16.4 (14.7-18.1)	16.6 (14.6-18.8)	16.3 (14.7-17.9)	.19
Total triiodothyronine (nmol/L)	1.83 (1.6-2.06)	1.83 (1.56-2.1)	1.83 (1.61-2.05)	.97
Total thyroxine (nmol/L)	94 (82.4-105)	93.9 (82.1-105)	94.2 (82.8-105)	.82
Alanine aminotransferase (U/L)	9 (7-14)	9 (7-14)	9 (7-14)	.97
Aspartate aminotransferase (U/L)	14 (12-17)	14.5 (12.8-17)	14 (12-17)	.91
Aspartate aminotransferase-to-alanine aminotransferase ratio	1.6 (1.2-1.9)	1.6 (1.2-2)	1.6 (1.12-1.9)	.95
Total protein (g/L)	67.7 (64.7-71.2)	68.5 (64.9-72.2)	67.3 (64.7-70.8)	.16
Albumin (g/L)	44.1 (42.3-46.2)	44.3 (42.6-47)	44 (42.2-45.8)	.12
Globulin (g/L)	23.5 (21.7-25.9)	23.7 (21.9-26.1)	23.5 (21.6-25.7)	.31
Albumin-to-globulin ratio	1.88 (1.7-2.06)	1.89 (1.71-2.03)	1.88 (1.7-2.07)	.93
Prealbumin (mg/L)	231 (205-268)	232 (204-272)	230 (205-263)	.45
Total bilirubin (µmol/L)	7.35 (5.1-11)	8 (5.6-11.9)	7.2 (5-10.5)	.07
Direct bilirubin (µmol/L)	3 (2.23-4.1)	3.2 (2.38-4.33)	2.9 (2.2-3.9)	.08
Indirect bilirubin (µmol/L)	4.35 (2.9-6.67)	4.8 (3.27-7.67)	4.15 (2.8-6.4)	.07
Alkaline phosphatase (U/L)	99 (79-135)	95.5 (78.8-134)	102 (79-136)	.35
Gamma-glutamyl transferase (U/L)	12 (9-16)	12 (10-16)	12 (9-16)	.25
Alpha-L-fucosidase (U/L)	17.2 (14.1-20.6)	17.5 (14.1-21.1)	17.1 (14.2-20)	.56
Fasting blood glucose (mmol/L)	4.71 (4.46-4.95)	4.71 (4.46-4.96)	4.71 (4.46-4.94)	.79
Total bile acid (µmol/L)	3 (1.8-5.17)	3.4 (2-5.9)	2.8 (1.8-4.7)	.06
Fructosamine (mmol/L)	1.98 (1.87-2.13)	2.04 (1.92-2.17)	1.97 (1.86-2.11)	*.009* ^b^
Blood urea nitrogen (mmol/L)	3.9 (3.3-4.7)	4 (3.38-4.83)	3.9 (3.2-4.6)	.15
Creatinine (µmol/L)	60 (53-67)	63 (54-72)	59 (52-65)	*.001*
Uric acid (µmol/L)	302 (255-358)	306 (262-374)	302 (253-348)	.23
Triglycerides (mmol/L)	0.96 (0.74-1.27)	0.99 (0.75-1.36)	0.94 (0.725-1.22)	.30
Total cholesterol (mmol/L)	3.93 (3.46-4.39)	3.97 (3.45-4.47)	3.91 (3.47-4.38)	.72
High-density lipoprotein cholesterol (mmol/L)	1.19 (1.06-1.37)	1.17 (1.05-1.36)	1.19 (1.07-1.37)	.48
Low-density lipoprotein cholesterol (mmol/L)	2.3 (1.94-2.73)	2.3 (1.94-2.73)	2.29 (1.94-2.73)	.95
Apolipoprotein A (g/L)	1.28 (1.14-1.47)	1.27 (1.14-1.47)	1.28 (1.14-1.46)	.76
Apolipoprotein B (g/L)	0.73 (0.63-0.87)	0.755 (0.65-0.885)	0.72 (0.62-0.867)	.13
Lipoprotein(a) (mg/L)	85.9 (43.1-244)	74.7 (36-253)	90.2 (46-226)	.37
Creatine kinase (U/L)	66.5 (53-88)	76 (55-94.8)	64.5 (51-82)	*.005*
Lactate dehydrogenase (U/L)	155 (141-171)	158 (143-174)	154 (140-170)	.09
Homocysteine (µmol/L)	10 (8-13)	10 (8-14)	10 (8-13)	.14
Potassium (mmol/L)	4.01 (3.86-4.2)	4.04 (3.87-4.2)	4 (3.86-4.2)	.40
Sodium (mmol/L)	139 (138-141)	139 (138-141)	139 (138-141)	.68
Chloride (mmol/L)	104 (103-106)	104 (103-106)	104 (103-105)	.60
Calcium (mmol/L)	2.31 (2.26-2.38)	2.31 (2.26-2.39)	2.31 (2.26-2.38)	.78
Magnesium (mmol/L)	0.87 (0.83-0.92)	0.88 (0.84-0.92)	0.87 (0.83-0.91)	.19
Phosphorus (mmol/L)	1.48 (1.37-1.6)	1.49 (1.37-1.61)	1.48 (1.38-1.59)	.59
Serum iron (µmol/L)	16.3 (11.7-21.2)	16.6 (12.4-21)	16.2 (11.5-21.2)	.46
High-sensitivity C-reactive protein (mg/L)	0.27 (0.16-0.6)	0.32 (0.177-0.535)	0.25 (0.15-0.66)	.19
Complement C3 (g/L)	0.92 (0.82-1.04)	0.92 (0.79-1.04)	0.92 (0.83-1.04)	.57
Complement C4 (g/L)	0.17 (0.14-0.21)	0.17 (0.14-0.21)	0.17 (0.13-0.22)	.74
Amylase (U/L)	61 (49-74)	62 (49-76.5)	61 (49-74)	.45
Prolactin (ng/mL)	25.8 (16.3-39)	23.2 (15.4-37)	26.9 (17.3-39.7)	.11

^a^NSSI: nonsuicidal self-injury.

^b^Italicized values are statistically significant (*P*<.05).

**Table 3:** Clinical and behavioral characteristics according to past-year NSSI^a^ status.

Characteristic		Total sample (N=410)	Non-NSSI (n=136)	NSSI (n=274)	*P* value
**Psychotic-symptom category, n (%)**					.04
	No psychotic symptoms	134 (32.7)	34 (25.0)	100 (36.5)	
	Psychotic symptoms present	200 (48.8)	77 (56.6)	123 (44.9)	
	Other/uncategorized category	76 (18.5)	25 (18.4)	51 (18.6)	
**Depression-grade category, n (%)**					.52
	Other/uncategorized category	24 (5.9)	6 (4.4)	18 (6.6)	
	Mild depression	3 (0.7)	2 (1.5)	1 (0.4)	
	Moderate depression	31 (7.6)	10 (7.4)	21 (7.7)	
	Severe depression	352 (85.9)	118 (86.8)	234 (85.4)	
Duration of illness (months), median (IQR)		12 (8-24)	12 (6-24)	12 (8.25-24)	.08
**First episode, n (%)**					.11
	No	298 (72.7)	92 (67.6)	206 (75.2)	
	Yes	112 (27.3)	44 (32.4)	68 (24.8)	
**Suicidal ideation, n (%)**					<.001
	No	123 (30.0)	70 (51.5)	53 (19.3)	
	Yes	287 (70.0)	66 (48.5)	221 (80.7)	
**Previous suicide attempt, n (%)**					<.001
	No	302 (73.7)	116 (85.3)	186 (67.9)	
	Yes	108 (26.3)	20 (14.7)	88 (32.1)	
**Verbal aggression, n (%)**					.26
	No	357 (87.1)	122 (89.7)	235 (85.8)	
	Yes	53 (12.9)	14 (10.3)	39 (14.2)	
**Physical aggression, n (%)**					.62
	No	392 (95.6)	131 (96.3)	261 (95.3)	
	Yes	18 (4.4)	5 (3.7)	13 (4.7)	

^a^NSSI: nonsuicidal self-injury.

### Model Development and Held-Out Test-Set Performance

In training-set cross-validation, the SVM with a radial basis function kernel achieved the highest mean AUROC (mean AUROC 0.720, SD 0.046). This was followed by logistic regression (mean AUROC 0.716, SD 0.049), SVM linear (mean AUROC 0.716, SD 0.025), decision tree (mean AUROC 0.709, SD 0.054), random forest (mean AUROC 0.703, SD 0.050), SVM sigmoid (mean AUROC 0.702, SD 0.062), and SVM polynomial (mean AUROC 0.680, SD 0.037). The relatively small differences in mean AUROC and overlapping cross-validation variability did not indicate clear separation in model performance during development. Detailed cross-validation results and optimized hyperparameters for all evaluated models are provided in Table S1 in Multimedia Appendix 1.

In the untouched held-out test set, the random forest had the highest observed AUROC and AUPRC among the 7 evaluated machine learning models (Table 4). Its AUROC was 0.681 (95% CI 0.583-0.778), and its AUPRC was 0.808 (95% CI 0.741-0.874). At the prespecified probability threshold of 0.50, the random forest had an accuracy of 0.667 (95% CI 0.585-0.748), sensitivity of 0.720 (95% CI 0.622-0.817), specificity of 0.561 (95% CI 0.415-0.707), positive predictive value of 0.766 (95% CI 0.703-0.835), negative predictive value of 0.500 (95% CI 0.395-0.622), and *F*_1_-score of 0.742 (95% CI 0.671-0.810). The corresponding confusion matrix contained 59 true-positive, 23 false-negative, 23 true-negative, and 18 false-positive classifications.

**Table 4:** Performance of the 7 machine learning models in the untouched held-out test set.^a^

Model	AUROC^b^ (95% CI)	AUPRC^c^ (95% CI)	Accuracy (95% CI)	Sensitivity (95% CI)	Specificity (95% CI)	PPV^d^ (95% CI)	NPV^e^ (95% CI)	*F*_1_-score (95% CI)	Brier score (95% CI)
Random forest	0.681 (0.583-0.778)	0.808 (0.741-0.874)	0.667 (0.585-0.748)	0.720 (0.622-0.817)	0.561 (0.415-0.707)	0.766 (0.703-0.835)	0.500 (0.395-0.622)	0.742 (0.671-0.810)	0.212 (0.188-0.236)
Decision tree	0.647 (0.552-0.737)	0.752 (0.696-0.811)	0.642 (0.561-0.724)	0.646 (0.537-0.744)	0.634 (0.488-0.780)	0.779 (0.708-0.852)	0.473 (0.385-0.566)	0.707 (0.621-0.778)	0.237 (0.202-0.276)
Logistic regression	0.620 (0.512-0.726)	0.730 (0.659-0.826)	0.626 (0.537-0.707)	0.659 (0.561-0.756)	0.561 (0.415-0.707)	0.750 (0.677-0.825)	0.451 (0.349-0.553)	0.701 (0.623-0.773)	0.242 (0.211-0.274)
SVM^f^-RBF^g^	0.619 (0.517-0.722)	0.746 (0.672-0.840)	0.650 (0.593-0.707)	0.890 (0.817-0.951)	0.171 (0.073-0.293)	0.682 (0.649-0.720)	0.438 (0.214-0.684)	0.772 (0.729-0.812)	0.219 (0.195-0.245)
SVM sigmoid	0.617 (0.510-0.722)	0.756 (0.683-0.838)	0.610 (0.561-0.659)	0.890 (0.817-0.951)	0.049 (0.000-0.122)	0.652 (0.627-0.675)	0.182 (0.000-0.444)	0.753 (0.713-0.786)	0.218 (0.200-0.237)
SVM linear	0.613 (0.504-0.722)	0.716 (0.651-0.814)	0.667 (0.602-0.732)	0.878 (0.805-0.951)	0.244 (0.122-0.390)	0.699 (0.660-0.743)	0.500 (0.294-0.714)	0.778 (0.731-0.823)	0.223 (0.194-0.252)
SVM polynomial	0.577 (0.465-0.684)	0.715 (0.646-0.811)	0.659 (0.602-0.715)	0.902 (0.829-0.963)	0.171 (0.073-0.293)	0.685 (0.652-0.721)	0.467 (0.222-0.714)	0.779 (0.738-0.817)	0.224 (0.202-0.247)

^a^All metrics were evaluated in the same held-out test set (n=123) at a probability threshold of 0.50, except AUROC, AUPRC, and Brier score, which are threshold-independent. CIs were obtained from 2000 stratified bootstrap resamples.

^b^AUROC: area under the receiver operating characteristic curve.

^c^AUPRC: area under the precision-recall curve.

^d^PPV: positive predictive value.

^e^NPV: negative predictive value.

^f^SVM: support vector machine.

^g^RBF: radial basis function kernel.

The decision tree showed the second-highest observed test-set AUROC at 0.647, followed by logistic regression (0.620), support vector machine with a radial basis function kernel (SVM-RBF; 0.619), SVM sigmoid (0.617), SVM linear (0.613), and SVM polynomial (0.577). Several SVM variants had higher sensitivity but lower specificity at the 0.50 threshold. Sensitivity ranged from 0.878 to 0.902 among the 4 SVM models, whereas specificity ranged from 0.049 to 0.244. In comparison, the random forest had a sensitivity of 0.720 and specificity of 0.561 at the same threshold.

Although the random forest showed numerically higher test-set AUROCs than the alternative models, none of the 6 pairwise comparisons remained statistically significant after Holm correction (Holm-adjusted *P* values ranged from .17 to .81). The largest observed difference was between the random forest and SVM polynomial (AUROC difference 0.104, 95% CI 0.011-0.197; unadjusted *P*=.03; Holm-adjusted *P*=.17). The difference between the random forest and the SVM-RBF model, which achieved the highest cross-validation AUROC during model development, was 0.062 (95% CI –0.027 to 0.152; unadjusted *P*=.17; Holm-adjusted *P*=.81). Therefore, the random forest was selected as the focal exploratory model for subsequent interpretation based on its overall held-out test-set performance and suitability for nonlinear interpretation; these comparisons did not demonstrate statistical superiority. Full pairwise AUROC comparisons between the random forest and alternative models are presented in Table S2 in Multimedia Appendix 1.

### Calibration and Decision-Curve Analysis

Calibration performance of the random forest model is summarized in Table 4 and illustrated in Figure 2B. The model achieved a Brier score of 0.212 (95% CI 0.188-0.236), with a calibration intercept of 0.457 and a calibration slope of 1.013. The calibration slope was close to the ideal value of 1, indicating that the spread of predicted probabilities was generally consistent with the observed outcomes. However, the positive calibration intercept suggested that the model tended to underestimate the overall observed probability of past-year NSSI. Across the 6 calibration groups, mean predicted probabilities ranged from 0.343 to 0.767, whereas the corresponding observed NSSI proportions ranged from 0.429 to 0.905.

**Figure 2:**
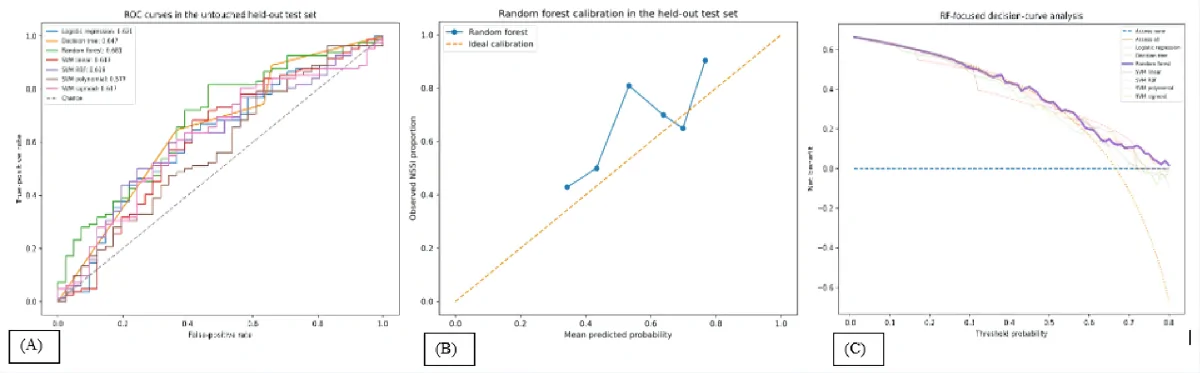
Performance evaluation and clinical utility analysis of the machine learning models. (A) Receiver operating characteristic (ROC) curves for the 7 model variants in the untouched held-out test set. (B) Calibration plot for the focal random forest model. (C) Decision curve analysis, with the random forest model highlighted. The modeled clinical action was referral for a comprehensive, structured nonsuicidal self-injury (NSSI) assessment rather than automatic diagnosis or treatment. RBF: radial basis function kernel; SVM: support vector machine.

Decision-curve analysis of the random forest model is shown in Figure 2C. The random forest demonstrated greater net benefit than both the assess-all and assess-none strategies across the main continuous threshold-probability range of approximately 0.51-0.80. At the prespecified threshold probability of 0.50, the net benefit of the random forest was approximately equivalent to that of the assess-all strategy. Compared with the other evaluated models, the random forest had the highest model-specific net benefit only within selected threshold intervals rather than across the entire probability range. These intervals included 0.01-0.10, 0.12-0.26, 0.34, 0.37-0.38, 0.45-0.46, 0.50-0.52, 0.56, 0.72-0.73, 0.76-0.77, and 0.79-0.80. Overall, the random forest had the highest model-specific net benefit at 40 of 80 (50%) evaluated threshold probabilities, indicating potential usefulness for identifying adolescents who may benefit from further structured NSSI assessment rather than serving as a standalone diagnostic tool.

### RF Incremental-Value and Sensitivity Analyses

Incremental-value and sensitivity analyses of the focal random forest model are summarized in Table 5. The suicidal-ideation-only logistic regression benchmark achieved an AUROC of 0.701 (95% CI 0.616-0.780), which was numerically higher than the AUROC of 0.681 for the full multidimensional random forest model. However, the difference between the full random forest and the suicidal-ideation-only benchmark was not statistically significant (AUROC difference –0.020, 95% CI –0.079 to 0.038). A benchmark model including both suicidal ideation and previous suicide attempt achieved an AUROC of 0.697 (95% CI 0.594-0.790).

**Table 5:** Incremental-value and sensitivity analyses of the focal random forest model.

Analysis	AUROC^a^ (95% CI)	AUPRC^b^	Accuracy	Sensitivity	Specificity	Brier score
SI^c^-only logistic benchmark	0.701 (0.616-0.780)	0.774	0.732	0.793	0.610	0.228
SI + previous-attempt logistic benchmark	0.697 (0.594-0.790)	0.770	0.732	0.793	0.610	0.226
RF^d^: basic clinical model	0.676 (0.559-0.778)	0.773	0.642	0.646	0.634	0.228
RF: clinical variables only	0.682 (0.573-0.781)	0.788	0.642	0.659	0.610	0.224
RF: full multidimensional model	0.681 (0.581-0.778)	0.808	0.667	0.720	0.561	0.212
RF: without SI and SA^e^	0.540 (0.429-0.655)	0.717	0.659	0.829	0.317	0.233
RF: biochemical variables only	0.513 (0.409-0.622)	0.685	0.618	0.793	0.268	0.238
RF: family/context variables only	0.488 (0.396-0.579)	0.661	0.455	0.390	0.585	0.253
RF: without sex	0.692 (0.597-0.785)	0.825	0.667	0.720	0.561	0.213
RF: without residence	0.678 (0.582-0.776)	0.808	0.667	0.732	0.537	0.214
RF: without FH^f^ and residence	0.676 (0.570-0.774)	0.806	0.659	0.707	0.561	0.213
RF: including length of stay	0.677 (0.570-0.776)	0.805	0.675	0.732	0.561	0.214
RF: training-only SMOTENC^g^ sensitivity	0.697 (0.593-0.798)	0.805	0.675	0.707	0.610	0.212

^a^AUROC: area under the receiver operating characteristic curve.

^b^AUPRC: area under the precision-recall curve.

^c^SI: suicidal ideation.

^d^RF: random forest.

^e^SA: suicide attempt.

^f^FH: family psychiatric history.

^g^SMOTENC: Synthetic Minority Over-Sampling Technique for Nominal and Continuous.

Removing suicidality-related variables reduced model discrimination. The random forest without suicidal ideation and previous suicide attempt achieved an AUROC of 0.540 (95% CI 0.429-0.655), compared with 0.681 for the full model (AUROC difference 0.141, 95% CI 0.045-0.237; Holm-adjusted *P*=.048). This finding indicated that suicidality-related variables contributed substantially to the full model’s discrimination of concurrent NSSI status in this sample, while also highlighting the close conceptual relationship between NSSI and suicidality-related clinical features.

Domain-specific sensitivity analyses showed that the clinical-only random forest achieved an AUROC of 0.682 (95% CI 0.573-0.781), which was comparable with that of the full multidimensional model (AUROC difference –0.001). The basic clinical random forest achieved an AUROC of 0.676 (95% CI 0.559-0.778). By contrast, the biochemical-only model achieved an AUROC of 0.513 (95% CI 0.409-0.622), and the available family/context-only model achieved an AUROC of 0.488 (95% CI 0.396-0.579). The full random forest model showed higher discrimination than the biochemical-only and available family/context-only models after Holm correction (adjusted *P*=.048 and .03, respectively). These findings did not demonstrate measurable incremental discrimination from adding the available biochemical variables to the clinical features.

Sensitivity analyses addressing potential demographic and contextual influences showed minimal changes in model performance. Excluding sex resulted in an AUROC of 0.692, excluding residence resulted in an AUROC of 0.678, and excluding both family psychiatric history and residence resulted in an AUROC of 0.676. Adding completed length of stay also did not improve discrimination (AUROC 0.677). These results indicated that excluding sex, residence, available family-context variables, or completed length of stay had limited effects on model discrimination in our sample.

In the class-imbalance sensitivity analysis, SMOTENC was applied exclusively within the training data. The resulting random forest achieved an AUROC of 0.697 (95% CI 0.593-0.798), an AUPRC of 0.805 (95% CI 0.735-0.879), an accuracy of 0.675, sensitivity of 0.707, specificity of 0.610, and Brier score of 0.212. The calibration slope was 0.959. These results were broadly consistent with those of the primary class-weighted random forest model, indicating that the observed model performance was not substantially altered by the approach used to address class imbalance. The AUROC distributions of these incremental-value and ablation analyses, with bootstrap CIs, are additionally presented in Figure S1 in Multimedia Appendix 1.

### Sex- and Residence-Specific Performance

Exploratory subgroup analyses according to sex and residence are summarized in Table S3 in Multimedia Appendix 1. Among male participants in the held-out test set (n=26; 15 with NSSI and 11 without NSSI), the random forest had an AUROC of 0.776 (95% CI 0.576-0.945). At the 0.50 threshold, sensitivity was 0.267 and specificity was 1.000. Among female participants (n=97; 67 with NSSI and 30 without NSSI), the AUROC was 0.665 (95% CI 0.548-0.777), sensitivity was 0.821, and specificity was 0.400. The wide CI among male participants and the markedly different sensitivity-specificity profiles indicated substantial uncertainty in the subgroup-specific performance estimates.

Among participants coded as urban residents in the held-out test set (n=74; 50 with NSSI and 24 without NSSI), the random forest had an AUROC of 0.718 (95% CI 0.587-0.835), sensitivity of 0.660, and specificity of 0.667. Among participants coded as rural residents (n=49; 32 with NSSI and 17 without NSSI), the AUROC was 0.619 (95% CI 0.445-0.778), sensitivity was 0.813, and specificity was 0.412. The CIs overlapped, and removal of residence from the full model resulted in minimal change in overall AUROC. Hospital-specific performance and leave-one-hospital-out validation could not be evaluated because the processed analytic dataset did not contain a hospital-site identifier.

### Random Forest Model Interpretation

According to Gini impurity–based importance, the 5 highest-ranked variables in the focal random forest model were suicidal ideation, sex, creatinine, T3, and TSH. Their corresponding Gini importance values were 0.157, 0.096, 0.032, 0.030, and 0.028, respectively. Previous suicide attempt ranked 10th, high-sensitivity C-reactive protein ranked 12th, and age ranked 17th among the 67 candidate variables. All candidate variables remained included in the fitted random forest model; Figure 3 displays the 20 highest-ranked variables according to Gini importance for visual clarity. Gini importance reflects the contribution of each variable to impurity reduction within the fitted tree ensemble and should not be interpreted as evidence of a causal relationship.

**Figure 3:**
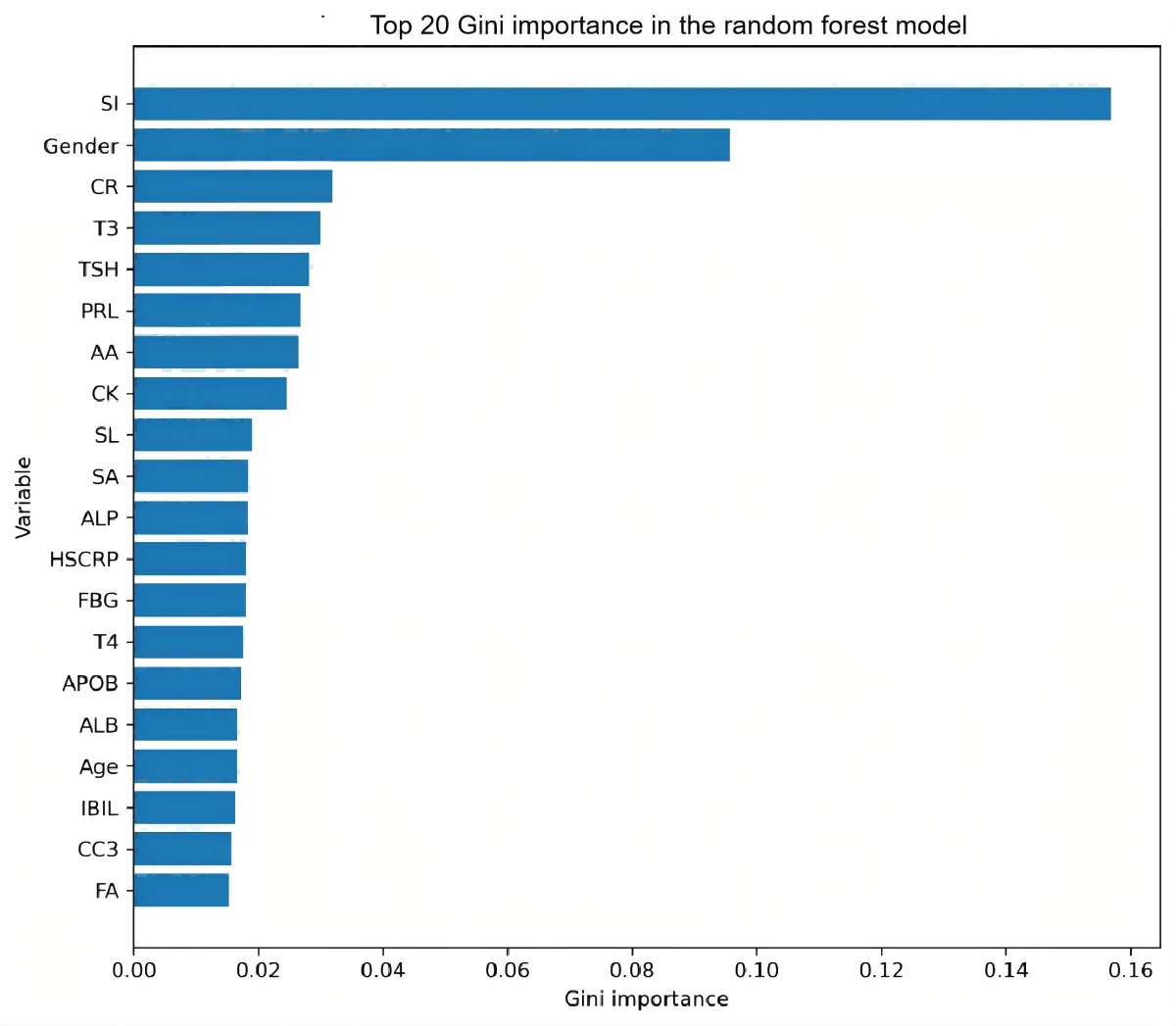
The 20 highest-ranked variables according to Gini importance in the focal random forest model. The fitted random forest included all 67 candidate variables; only the 20 highest-ranked variables are shown for visual clarity. AA: amylase; ALB: albumin; ALP: alkaline phosphatase; APOB: apolipoprotein B; CC3: complement component C3; CK: creatine kinase; CR: creatinine; FA: free fatty acid; FBG: fasting blood glucose; HSCRP: high-sensitivity C-reactive protein; IBIL: indirect bilirubin; PRL: prolactin; SA: suicide attempt; SI: self-injury; SL: aspartate aminotransferase-to-alanine aminotransferase ratio; T3: total triiodothyronine; T4: total thyroxine; TSH: thyroid-stimulating hormone.

Mean absolute SHA*P* values showed a broadly similar ranking pattern. Suicidal ideation, sex, creatinine, T3, and TSH were again among the 5 highest-ranked variables, followed by previous suicide attempt, creatine kinase, prolactin, age, and the aspartate aminotransferase-to-alanine aminotransferase ratio. The Gini importance and mean absolute SHAP rankings were strongly correlated (Spearman ρ=0.917, *P*<.001), showing substantial agreement between the 2 model-specific importance measures. A detailed comparison of variable rankings across Gini importance, SHA*P* values, and permutation importance is provided in Table S4 in Multimedia Appendix 1. However, differences in individual rankings were observed, reflecting that Gini importance and SHA*P* values capture different aspects of variable contribution within tree-based models.

Test-set permutation importance provided a more conservative assessment of feature contribution on unseen data. Permuting suicidal ideation resulted in the largest reduction in test-set AUROC (mean decrease 0.134), which was substantially greater than the reductions associated with amylase (mean decrease 0.014), age (mean decrease 0.006), previous suicide attempt (mean decrease 0.005), free thyroxine (mean decrease 0.004), and creatine kinase (mean decrease 0.004). Although sex, T3, and TSH ranked highly according to Gini importance and SHA*P* values, their mean permutation importance values were negative (–0.002, –0.011, and –0.003, respectively; Figure 4). High-sensitivity C-reactive protein ranked 12th according to Gini importance and 20th according to SHA*P* values but also showed negative permutation importance. These findings indicated that suicidal ideation showed the most consistent model-specific contribution across the interpretation approaches, whereas the apparent contribution of several physiological variables varied depending on the interpretation method.

The SHAP beeswarm plot further characterized the direction and distribution of variable contributions within the fitted random forest model (Figure 5). The presence of suicidal ideation generally showed positive SHA*P* values, indicating a shift in model output toward classification as NSSI=1. Female sex also tended to contribute positively toward the NSSI class. In the continuous-feature dependence analyses, feature values were inversely correlated with SHAP contributions for creatinine (Spearman ρ=–0.909), T3 (Spearman ρ=–0.758), TSH (Spearman ρ=–0.901), age (Spearman ρ=–0.896), and creatine kinase (Spearman ρ=–0.692), whereas prolactin values showed a positive correlation with SHAP contributions (Spearman ρ=0.767). Within the fitted model, lower creatinine, T3, TSH, age, and creatine kinase values tended to shift predictions toward NSSI classification, whereas higher prolactin values tended to shift predictions toward NSSI classification. The creatine kinase pattern was interpreted cautiously because its distribution in the held-out test set was heterogeneous (range 24-3935 U/L). These SHAP-based patterns describe fitted-model behavior and should not be interpreted as causal or prospective effects.

**Figure 4:**
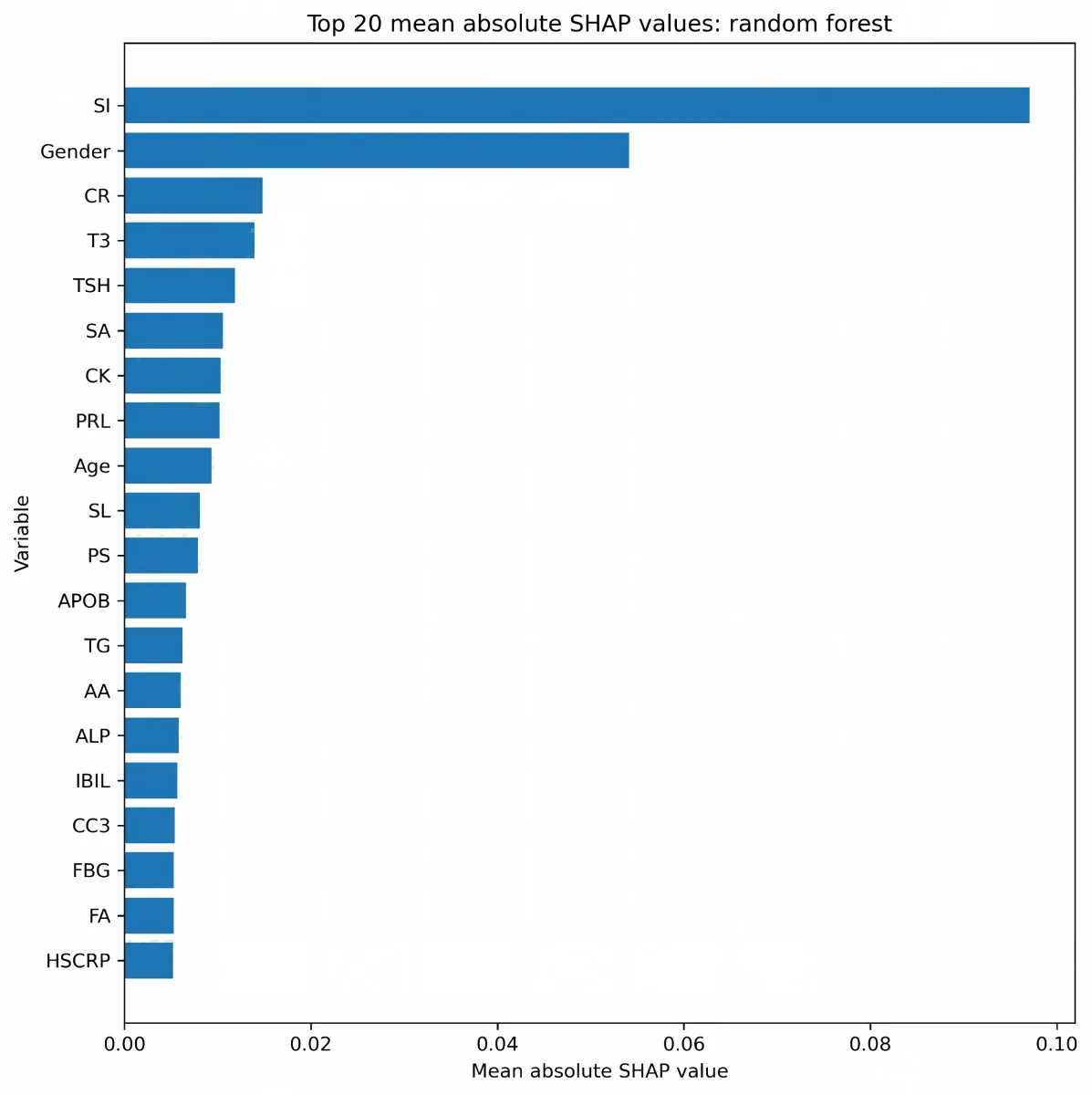
Mean absolute Shapley Additive Explanations (SHAP) values for the 20 highest-ranked variables in the focal random forest model. Mean absolute SHAP values quantify the magnitude of each variable's contribution to the model output but do not indicate the direction of the association. AA: amylase; ALP: alkaline phosphatase; APOB: apolipoprotein B; CC3: complement component C3; CK: creatine kinase; CR: creatinine; FA: free fatty acid; FBG: fasting blood glucose; HSCRP: high-sensitivity C-reactive protein; IBIL: indirect bilirubin; PRL: prolactin; PS: previous suicide attempt; SA: suicide attempt; SI: self-injury; SL: aspartate aminotransferase-to-alanine aminotransferase ratio; T3: total triiodothyronine; T4: total thyroxine; TG: triglyceride; TSH: thyroid-stimulating hormone.

**Figure 5:**
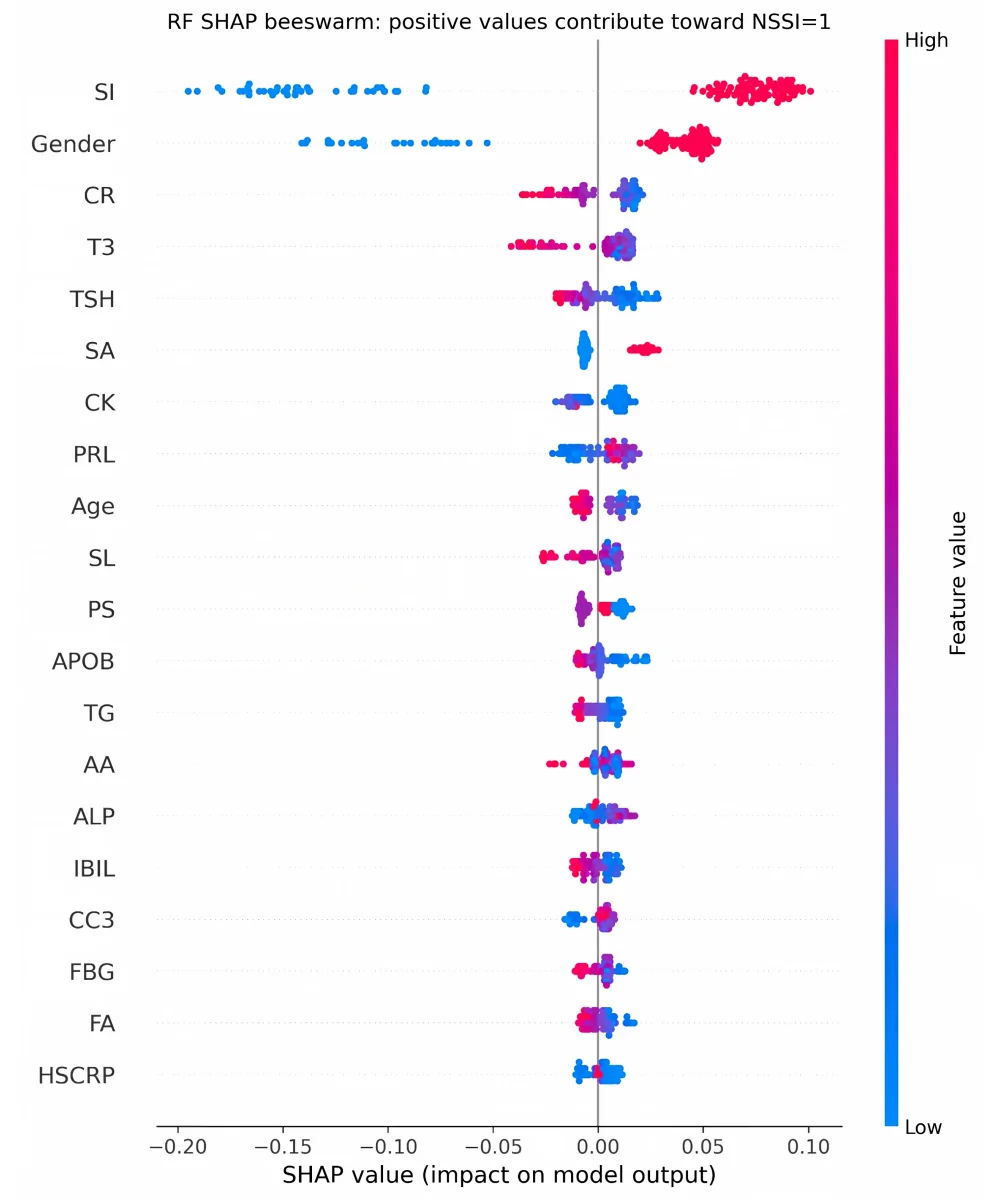
Shapley Additive Explanations (SHAP) beeswarm plot for the 20 highest-ranked variables in the focal random forest model. Positive SHAP values indicate contributions toward classification as nonsuicidal self-injury (NSSI)=1. Red points represent higher feature values, and blue points represent lower feature values. SHAP values describe the fitted model output and should not be interpreted as causal effects. AA: amylase; ALP: alkaline phosphatase; APOB: apolipoprotein B; CC3: complement component C3; CK: creatine kinase; CR: creatinine; FA: free fatty acid; FBG: fasting blood glucose; HSCRP: high-sensitivity C-reactive protein; IBIL: indirect bilirubin; PRL: prolactin; PS: previous suicide attempt; SA: suicide attempt; SI: self-injury; SL: aspartate aminotransferase-to-alanine aminotransferase ratio; T3: total triiodothyronine; TG: triglyceride; TSH: thyroid-stimulating hormone.

## Discussion

### Principal Findings

In this study, we evaluated 7 supervised machine learning models for classifying past-year NSSI status among adolescents with depressive disorders using multidimensional clinical, behavioral, and biochemical variables. The support vector machine with a radial basis function kernel achieved the highest mean AUROC during training-set cross-validation, whereas the random forest had the highest observed AUROC and AUPRC and the lowest Brier score in the untouched held-out test set among the evaluated models. However, the random forest did not demonstrate statistically significant superiority over alternative algorithms after correction for multiple comparisons. The discrepancy between training-set and held-out test-set rankings suggests that algorithm rankings may be unstable in relatively small clinical datasets.

Previous applications of machine learning to suicidal and self-injurious outcomes have demonstrated the potential of these approaches for classification and prediction [40]. However, systematic reviews have also identified methodological limitations, including inconsistent reporting and a lack of validation in external samples [41]. Recent longitudinal work has applied machine learning to identify clinical predictors of persistent NSSI trajectories among adolescents [37], while other NSSI-focused studies have reported favorable internal classification performance using machine learning algorithms [42]. Nevertheless, independent validation remains essential before such models can be considered for clinical implementation. Transparent reporting of model development and validation is important for evaluating the reliability and reproducibility of clinical prediction research [43], while careful assessment of risk of bias and applicability is required to identify potential methodological limitations [44]. The most consistent contributor to discrimination in the focal random forest model was suicidal ideation. The suicidal-ideation-only logistic regression benchmark achieved discrimination comparable to that of the random forest, whereas removal of suicidal ideation and previous suicide attempt substantially reduced random forest performance. These findings indicate that direct suicidality-related information accounted for a considerable proportion of the model’s classification ability in this sample. Longitudinal evidence has shown that adolescents engaging in NSSI or nonsuicidal self-harm are at increased risk of subsequent suicide attempts [45]. Prospective evidence further suggests that the co-occurrence of NSSI and suicidal ideation may identify adolescents at particularly elevated risk of subsequent suicide attempts [46]. Nevertheless, NSSI is conceptually distinguished from suicidal behavior by the absence of suicidal intent [47]. NSSI may serve multiple psychological and interpersonal functions, including regulation of negative affect and other intrapersonal or interpersonal functions [48]. Real-time monitoring studies further demonstrate substantial between-person and within-person variability in the functions of NSSI over time [49]. Person-centered analyses likewise support substantial heterogeneity in NSSI presentations and behavioral patterns [50].

Therefore, the prominent contribution of suicidal ideation in our model should not be interpreted as indicating that NSSI can be reduced to suicidality-related variables. Rather, our findings reinforce the importance of directly assessing suicidal ideation and previous suicidal behavior while maintaining a separate and comprehensive assessment of NSSI characteristics. In clinical practice, models incorporating suicidality-related information could potentially help organize complex patient information and identify adolescents who may warrant more comprehensive assessment, but they should complement rather than replace clinical judgment.

By contrast, biochemical variables did not demonstrate clear incremental discrimination beyond the available clinical information. Although creatinine, T3, TSH, creatine kinase, and prolactin appeared among the higher-ranked variables according to Gini importance or SHA*P* values, their contributions were less consistent in permutation-based analyses. Previous research has identified differences in inflammatory and immune-related biomarkers between adolescents with depression and healthy controls, although findings remain heterogeneous [51]. Peripheral biological correlates have also been investigated in relation to suicidality among children and adolescents, but their clinical utility remains uncertain [52]. More specifically, emerging research has suggested a possible role of inflammatory processes in adolescent depression accompanied by NSSI [53]. Neuroendocrine mechanisms may also be relevant, with studies reporting associations between NSSI and variations in testosterone and cortisol levels [54]. Alterations in the hypothalamic-pituitary-thyroid axis have similarly been reported among adolescents with major depressive disorder and NSSI [55]. However, these findings remain insufficient to establish clinically reliable biomarkers. In this study, high-sensitivity C-reactive protein did not differ significantly between participants with and without NSSI despite appearing among the higher-ranked variables in some model-interpretation analyses. This discrepancy illustrates an important limitation of interpreting variable importance in complex machine learning models: a variable that contributes to model discrimination does not necessarily represent an independent biological mechanism or causal risk factor [56]. Explainability methods may provide information about model behavior, but they should not be assumed to ensure trustworthy patient-level decision support or to substitute for rigorous model validation [57]. Tree-based algorithms can capture nonlinear relationships, interactions, and conditional dependencies that may not be apparent in conventional univariable comparisons. Accordingly, the physiological variables identified in this study should be interpreted as contributors to fitted-model behavior rather than as established biomarkers or causal determinants of NSSI.

The random forest demonstrated acceptable calibration characteristics and potential clinical usefulness within selected threshold ranges in decision-curve analysis. However, these findings do not justify automated diagnosis, treatment selection, hospitalization decisions, or direct risk labeling of adolescents. Evaluation of clinical prediction models should extend beyond discrimination alone and include calibration, validation, and assessment of potential clinical usefulness [58]. Decision-curve analysis provides a framework for estimating net clinical benefit across different threshold probabilities [59]. Nevertheless, favorable decision-curve results cannot substitute for prospective evaluation of whether use of a model actually improves clinical outcomes. Similarly, although explainability methods can provide information about how a model generates its predictions, such explanations should not be assumed to ensure trustworthy patient-level decision support or to substitute for rigorous model validation [57]. Translation of AI from retrospective model development into clinical practice therefore requires staged and rigorous evaluation of real-world performance and clinical utility [60]. Trustworthy implementation also requires attention to robustness, fairness, transparency, usability, and potential unintended harms throughout the model life cycle [61]. Importantly, meaningful clinician oversight should remain central when AI is used to support decisions in complex and high-stakes psychiatric settings [62].

Another important consideration is the limited representation of family and broader social-contextual factors in the present model. Family dynamics have been consistently associated with self-harm and suicidality among children and adolescents [63]. Peer relationships, including interpersonal difficulties and social experiences, may also contribute meaningfully to the development and maintenance of self-harm thoughts and behaviors [64]. As the available dataset contained relatively limited information regarding family functioning, peer relationships, interpersonal stressors, social support, and adverse experiences, the model may not have fully captured important developmental and interpersonal pathways associated with NSSI. Future models may therefore benefit from incorporating more comprehensive psychosocial assessments alongside clinical and biological information. Standardized measures of family functioning, peer relationships, emotion regulation, adverse experiences, and social support could help determine whether these variables provide meaningful incremental value beyond readily available clinical indicators. In addition, clinical evaluation of AI systems should consider whether the information provided by a model is understandable, actionable, and compatible with real-world clinical workflows [60]. Broader frameworks for trustworthy health care AI also emphasize the importance of human-centered development and stakeholder involvement when models are intended for clinical use [61]. Involving adolescents, caregivers, and clinicians in future model development and evaluation may therefore improve the clinical relevance and acceptability of prediction tools.

Overall, our findings suggest that machine learning can integrate multidimensional clinical information to assist in identifying adolescents with depressive disorders who report past-year NSSI. However, the substantial contribution of suicidal ideation and previous suicidal behavior indicates that much of the model’s discriminatory ability was derived from established clinical indicators rather than from biochemical information. The comparable discrimination achieved by the suicidal-ideation-only benchmark further suggests that the incremental value of a more complex multidimensional model should be interpreted cautiously. Nevertheless, machine learning may provide a means of integrating heterogeneous information and capturing complex patterns that may not be readily identified using conventional approaches. Rather than replacing standard clinical assessment, interpretable machine learning may be most appropriately positioned as an adjunctive tool for organizing multidimensional information, identifying patterns that warrant further evaluation, and supporting comprehensive clinician-led assessment.

### Limitations

Several limitations warrant consideration. First, the sample size was relatively small and drawn from psychiatric clinical settings in Zhejiang Province, which may limit the generalizability of the findings. Second, the cross-sectional design precludes causal inferences; therefore, future longitudinal studies involving larger and more diverse populations are needed to externally validate and refine these models and to evaluate their prospective performance. Lastly, incorporating self-reported psychosocial data, such as family dynamics and peer relationships, alongside clinician-assessed information could provide a more comprehensive representation of the psychosocial factors relevant to NSSI.

### Implication

This study provides clinical and research implications for understanding the clinical characteristics associated with NSSI among adolescents with depressive disorders. By integrating demographic, physiological, biochemical, and behavioral variables, this study demonstrates the potential of combining multidimensional information to characterize clinical profiles associated with NSSI. The interpretable machine learning approach may assist in identifying adolescents with past-year NSSI and provide information to support comprehensive assessment. Importantly, the model should be considered a decision-support tool rather than a replacement for clinical judgment. Future studies incorporating larger samples, longitudinal designs, external validation, and richer psychosocial information are warranted to further evaluate the robustness, generalizability, and clinical applicability of such models. These findings may contribute to the development of more individualized assessment strategies and support the identification of adolescents who may benefit from comprehensive evaluation and targeted clinical support.

### Conclusions

This study demonstrates the potential utility of integrating multidimensional clinical, behavioral, and biochemical data with interpretable machine learning approaches to classify past-year NSSI status among adolescents with depressive disorders. The findings suggest that machine learning models integrating heterogeneous information may provide complementary support for comprehensive clinical assessment. However, further validation in larger and independent cohorts, particularly using longitudinal designs, is required before clinical implementation. Ultimately, interpretable machine learning models may serve as useful adjuncts to clinician-led assessment and may contribute to improving the identification and evaluation of adolescents with NSSI.

## Appendix

Multimedia Appendix 1 Additional analysis.

## Abbreviations

AUPRC: area under the precision-recall curve
AUROC: area under the receiver operating characteristic curve
DSM-5: Diagnostic and Statistical Manual of Mental Disorders, Fifth Edition
ICD-10: International Classification of Diseases, 10th Revision
NSSI: nonsuicidal self-injury
RF: random forest
SHAP: Shapley Additive Explanations
SMOTENC: Synthetic Minority Over-Sampling Technique for Nominal and Continuous
SVM-RBF: support vector machine with a radial basis function kernel
T3: total triiodothyronine
T4: total thyroxine
TSH: thyroid-stimulating hormone

## Data Availability

The data that support the findings of this study are available from the corresponding author (KZ) upon reasonable request. However, due to ethical and privacy restrictions, the data are not publicly accessible.

## References

[1] Thapar A, Eyre O, Patel V, Brent D (2022). Depression in young people. Lancet.

[2] Plener PL, Kaess M, Schmahl C, Pollak S, Fegert JM, Brown RC (2018). Nonsuicidal self-injury in adolescents. Dtsch Arztebl Int.

[3] Gillies D, Christou MA, Dixon AC, Featherston OJ, Rapti I, Garcia-Anguita A, Villasis-Keever M, Reebye P, Christou E, Al Kabir N, Christou PA (2018). Prevalence and characteristics of self-harm in adolescents: meta-analyses of community-based studies 1990-2015. J Am Acad Child Adolesc Psychiatry.

[4] Lim K, Wong CH, McIntyre RS, Wang J, Zhang Z, Tran BX, Tan W, Ho CS, Ho RC (2019). Global lifetime and 12-month prevalence of suicidal behavior, deliberate self-harm and non-suicidal self-injury in children and adolescents between 1989 and 2018: a meta-analysis. Int J Environ Res Public Health.

[5] Qu D, Wen X, Liu B, Zhang X, He Y, Chen D, Duan X, Yu J, Liu D, Zhang X, Ou J, Zhou J, Cui Z, An J, Wang Y, Zhou X, Yuan T, Tang J, Yue W, Chen R (2023). Non-suicidal self-injury in Chinese population: a scoping review of prevalence, method, risk factors and preventive interventions. Lancet Reg Health West Pac.

[6] He H, Hong L, Jin W, Xu Y, Kang W, Liu J, Song J, Zheng T, Chen H, Zhao K (2023). Heterogeneity of non-suicidal self-injury behavior in adolescents with depression: latent class analysis. BMC Psychiatry.

[7] Daukantaitė D, Lundh L, Wångby-Lundh M, Claréus B, Bjärehed J, Zhou Y, Liljedahl SI (2021). What happens to young adults who have engaged in self-injurious behavior as adolescents? A 10-year follow-up. Eur Child Adolesc Psychiatry.

[8] Ribeiro JD, Franklin JC, Fox KR, Bentley KH, Kleiman EM, Chang BP, Nock MK (2016). Self-injurious thoughts and behaviors as risk factors for future suicide ideation, attempts, and death: a meta-analysis of longitudinal studies. Psychol Med.

[9] Kiekens G, Hasking P, Boyes M, Claes L, Mortier P, Auerbach R, Cuijpers P, Demyttenaere K, Green J, Kessler R, Myin-Germeys I, Nock M, Bruffaerts R (2018). The associations between non-suicidal self-injury and first onset suicidal thoughts and behaviors. Journal of Affective Disorders.

[10] Tang S, Hoye A, Slade A, Tang B, Holmes G, Fujimoto H, Zheng W, Ravindra S, Christensen H, Calear AL (2025). Motivations for self-harm in young people and their correlates: a systematic review. Clin Child Fam Psychol Rev.

[11] Hepp J, Carpenter RW, Störkel LM, Schmitz SE, Schmahl C, Niedtfeld I (2020). A systematic review of daily life studies on non-suicidal self-injury based on the four-function model. Clin Psychol Rev.

[12] Rheinberger D, Ravindra S, Slade A, Calear AL, Wang A, Bunyan B, Christensen H, Mahony I, Gilbert I, Boydell K, Hankin L, Tang S (2025). Exploring support preferences for young women who self-harm: a qualitative study. Int J Environ Res Public Health.

[13] Pisani AR, Schmeelk-Cone K, Gunzler D, Petrova M, Goldston DB, Tu X, Wyman PA (2012). Associations between suicidal high school students' help-seeking and their attitudes and perceptions of social environment. J Youth Adolesc.

[14] Cha CB, Wilson KM, Tezanos KM, DiVasto KA, Tolchin GK (2019). Cognition and self-injurious thoughts and behaviors: a systematic review of longitudinal studies. Clin Psychol Rev.

[15] Walsh CG, Ribeiro JD, Franklin JC (2018). Predicting suicide attempts in adolescents with longitudinal clinical data and machine learning. J Child Psychol Psychiatry.

[16] van Mens K, de Schepper C, Wijnen B, Koldijk SJ, Schnack H, de Looff P, Lokkerbol J, Wetherall K, Cleare S, C O'Connor R, de Beurs D (2020). Predicting future suicidal behaviour in young adults, with different machine learning techniques: a population-based longitudinal study. J Affect Disord.

[17] Guo X, Liu S, Jiang L, Xiong Z, Wang L, Lu L, Li X, Zhao L, Shek DT (2026). Longitudinal machine learning prediction of non-suicidal self-injury among Chinese adolescents: a prospective multicenter Cohort study. J Affect Disord.

[18] Reichl C, Fink E, von den Driesch L, Lerch S, Koenig J, Berger T, Kaess M (2026). Integrating neurobiological markers to prospectively predict adolescent non-suicidal self-injury and suicide attempts: a machine learning approach. Child Adolesc Psychiatry Ment Health.

[19] Wu B, Zhang H, Chen J, Chen J, Liu Z, Cheng Y, Yuan T, Peng D (2023). Potential mechanisms of non-suicidal self-injury (NSSI) in major depressive disorder: a systematic review. Gen Psychiatr.

[20] Wolff JC, Thompson E, Thomas SA, Nesi J, Bettis AH, Ransford B, Scopelliti K, Frazier EA, Liu RT (2019). Emotion dysregulation and non-suicidal self-injury: a systematic review and meta-analysis. Eur Psychiatry.

[21] Bresin K, Schoenleber M (2015). Gender differences in the prevalence of nonsuicidal self-injury: a meta-analysis. Clinical Psychology Review.

[22] de Neve-Enthoven NGM, Ringoot AP, Jongerling J, Boersma N, Berges LM, Meijnckens D, Hoogendijk WJG, Grootendorst-van Mil NH (2024). Adolescent nonsuicidal self-injury and suicidality: a latent class analysis and associations with clinical characteristics in an at-risk cohort. J Youth Adolesc.

[23] DeVille DC, Whalen D, Breslin FJ, Morris AS, Khalsa SS, Paulus MP, Barch DM (2020). Prevalence and family-related factors associated with suicidal ideation, suicide attempts, and self-injury in children aged 9 to 10 years. JAMA Netw Open.

[24] He Y, Jiang W, Wang W, Liu Q, Peng S, Guo L (2024). Adverse childhood experiences and nonsuicidal self-injury and suicidality in Chinese adolescents. JAMA Netw Open.

[25] Lin Z, Zhang Y, Kong S, Ruan Q, Zhu L, Li C (2025). Social support as a mediator between life events and non-suicidal self-injury: evidence for urban-rural moderation in medical students. Front Psychiatry.

[26] Kindler J, Koenig J, Lerch S, van der Venne P, Resch F, Kaess M (2022). Increased immunological markers in female adolescents with non-suicidal self-injury. J Affect Disord.

[27] Ma J, Zhao M, Niu G, Wang Z, Jiang S, Liu Z (2022). Relationship between thyroid hormone and sex hormone levels and non-suicidal self-injury in male adolescents with depression. Front Psychiatry.

[28] Fox KR, Huang X, Linthicum KP, Wang SB, Franklin JC, Ribeiro JD (2019). Model complexity improves the prediction of nonsuicidal self-injury. J Consult Clin Psychol.

[29] Asarnow JR, Berk MS, Bedics J, Adrian M, Gallop R, Cohen J, Korslund K, Hughes J, Avina C, Linehan MM, McCauley E (2021). Dialectical behavior therapy for suicidal self-harming youth: emotion regulation, mechanisms, and mediators. Journal of the American Academy of Child & Adolescent Psychiatry.

[30] Nasarian E, Alizadehsani R, Acharya U, Tsui K (2024). Designing interpretable ML system to enhance trust in healthcare: a systematic review to proposed responsible clinician-AI-collaboration framework. Information Fusion.

[31] Zhong Y, He J, Luo J, Zhao J, Cen Y, Song Y, Wu Y, Lin C, Pan L, Luo J (2024). A machine learning algorithm-based model for predicting the risk of non-suicidal self-injury among adolescents in western China: a multicentre cross-sectional study. J Affect Disord.

[32] Wang Y, Lin J, Zhu Z, Chen S, Zou X, Wang Y, Huo L, Zhou Y (2025). Quantifying the importance of factors in predicting non-suicidal self-injury among depressive Chinese adolescents: a comparative study between only child and non-only child groups. Journal of Affective Disorders.

[33] Zhao Y, Wang Q, Liu W (2026). Development and validation of a machine learning-based risk prediction model for non-suicidal self-injury in adolescents. Front Psychiatry.

[34] Lee JO, Moon H, Zoh S, Jo E, Hur J (2024). Neural correlates of reward valuation in individuals with nonsuicidal self-injury under uncertainty. Psychol Med.

[35] Chekroud AM, Bondar J, Delgadillo J, Doherty G, Wasil A, Fokkema M, Cohen Z, Belgrave D, DeRubeis R, Iniesta R, Dwyer D, Choi K (2021). The promise of machine learning in predicting treatment outcomes in psychiatry. World Psychiatry.

[36] Lundberg SM, Erion G, Chen H, DeGrave A, Prutkin JM, Nair B, Katz R, Himmelfarb J, Bansal N, Lee S (2020). From local explanations to global understanding with explainable AI for trees. Nat Mach Intell.

[37] Mason GE, Auerbach RP, Stewart JG (2025). Predicting the trajectory of non-suicidal self-injury among adolescents. J Child Psychol Psychiatry.

[38] Vickers AJ, Van Calster B, Steyerberg EW (2016). Net benefit approaches to the evaluation of prediction models, molecular markers, and diagnostic tests. BMJ.

[39] Nembrini S, König IR, Wright MN (2018). The revival of the Gini importance?. Bioinformatics.

[40] Nordin N, Zainol Z, Mohd Noor MH, Chan LF (2022). Suicidal behaviour prediction models using machine learning techniques: a systematic review. Artif Intell Med.

[41] Burke TA, Ammerman BA, Jacobucci R (2019). The use of machine learning in the study of suicidal and non-suicidal self-injurious thoughts and behaviors: a systematic review. J Affect Disord.

[42] Huo X, Liu X, Wang Y, Yang L, Huang Y, Wang Y, Zheng H, Zhao X (2026). The prediction and impact of self-criticism on non-suicidal self-injury in adolescents: a study based on machine learning. Acta Psychol (Amst).

[43] Collins GS, Reitsma JB, Altman DG, Moons KG (2015). Transparent Reporting of a multivariable prediction model for Individual Prognosis or Diagnosis (TRIPOD): the TRIPOD statement. Ann Intern Med.

[44] Wolff RF, Moons KGM, Riley RD, Whiting PF, Westwood M, Collins GS, Reitsma JB, Kleijnen J, Mallett S, PROBAST Group† (2019). PROBAST: a tool to assess the risk of bias and applicability of prediction model studies. Ann Intern Med.

[45] Mars B, Heron J, Klonsky ED, Moran P, O'Connor RC, Tilling K, Wilkinson P, Gunnell D (2019). Predictors of future suicide attempt among adolescents with suicidal thoughts or non-suicidal self-harm: a population-based birth cohort study. Lancet Psychiatry.

[46] Scott LN, Pilkonis PA, Hipwell AE, Keenan K, Stepp SD (2015). Non-suicidal self-injury and suicidal ideation as predictors of suicide attempts in adolescent girls: a multi-wave prospective study. Compr Psychiatry.

[47] Klonsky ED (2007). The functions of deliberate self-injury: a review of the evidence. Clinical Psychology Review.

[48] Lloyd-Richardson EE, Perrine N, Dierker L, Kelley ML (2007). Characteristics and functions of non-suicidal self-injury in a community sample of adolescents. Psychol Med.

[49] Coppersmith DD, Bentley KH, Kleiman EM, Nock MK (2021). Variability in the functions of nonsuicidal self-injury: evidence from three real-time monitoring studies. Behavior Therapy.

[50] Shahwan S, Lau JH, Abdin E, Zhang Y, Sambasivam R, Teh WL, Gupta B, Ong SH, Chong SA, Subramaniam M (2020). A typology of nonsuicidal self-injury in a clinical sample: a latent class analysis. Clin Psychol Psychother.

[51] Li J, Zhang Y, Yang N, Du J, Liu P, Dai W, Dong Q (2025). Differences between adolescent depression and healthy controls in biomarkers associated with immune or inflammatory processes: a systematic review and meta-analysis. Psychiatry Investig.

[52] Pak TK, Ayvaci ER, Carmody T, Jamma L, Feng Z, Nekovei A, Emslie G, Trivedi MH (2025). Peripheral biological correlates of suicidality in children and adolescents: a systematic review and meta-analysis. iScience.

[53] Qiao D, Qi Y, Zhang X, Wen Y, Huang Y, Li Y, Liu P, Li G, Liu Z (2025). The possible effect of inflammation on non-suicidal self-injury in adolescents with depression: a mediator of connectivity within corticostriatal reward circuitry. Eur Child Adolesc Psychiatry.

[54] Calvete E, Prieto-Fildalgo A, Faura-García J, Orue I (2024). The role of testosterone and cortisol levels in nonsuicidal selfinjury in adolescents. J Adolesc.

[55] Zhan D, Jin T, Zhao H, Zhu H, Fu J, Zhang X, Sun W, Zhang X, Chen P (2026). Hypothalamic-pituitary-thyroid axis dysregulation in adolescents with major depressive disorder and non-suicidal self-injury: a retrospective study. J Affect Disord.

[56] Carriero A, de Hond A, Cappers B, Paulovich F, Abeln S, Moons KG, van Smeden M (2025). Explainable AI in healthcare: to explain, to predict, or to describe?. Diagn Progn Res.

[57] Ghassemi M, Oakden-Rayner L, Beam AL (2021). The false hope of current approaches to explainable artificial intelligence in health care. Lancet Digit Health.

[58] Binuya MAE, Engelhardt EG, Schats W, Schmidt MK, Steyerberg EW (2022). Methodological guidance for the evaluation and updating of clinical prediction models: a systematic review. BMC Med Res Methodol.

[59] Vickers AJ, Elkin EB (2006). Decision curve analysis: a novel method for evaluating prediction models. Med Decis Making.

[60] Vasey B, Nagendran M, Campbell B, Clifton DA, Collins GS, Denaxas S, Denniston AK, Faes L, Geerts B, Ibrahim M, Liu X, Mateen BA, Mathur P, McCradden MD, Morgan L, Ordish J, Rogers C, Saria S, Ting DSW, Watkinson P, Weber W, Wheatstone P, McCulloch P, DECIDE-AI expert group (2022). Reporting guideline for the early-stage clinical evaluation of decision support systems driven by artificial intelligence: DECIDE-AI. Nat Med.

[61] Lekadir K, Frangi AF, Porras AR, Glocker B, Cintas C, Langlotz CP, Weicken E, Asselbergs FW, Prior F, Collins GS, Kaissis G, Tsakou G, Buvat I, Kalpathy-Cramer J, Mongan J, Schnabel JA, Kushibar K, Riklund K, Marias K, Amugongo LM, Fromont LA, Maier-Hein L, Cerdá-Alberich L, Martí-Bonmatí L, Cardoso MJ, Bobowicz M, Shabani M, Tsiknakis M, Zuluaga MA, Fritzsche M, Camacho M, Linguraru MG, Wenzel M, De Bruijne M, Tolsgaard MG, Goisauf M, Cano Abadía M, Papanikolaou N, Lazrak N, Pujol O, Osuala R, Napel S, Colantonio S, Joshi S, Klein S, Aussó S, Rogers WA, Salahuddin Z, Starmans MPA, FUTURE-AI Consortium (2025). FUTURE-AI: international consensus guideline for trustworthy and deployable artificial intelligence in healthcare. BMJ.

[62] Kelly BD (2026). Human in the loop: balancing artificial intelligence, clinical judgment, and legal responsibility. Int J Law Psychiatry.

[63] Hammond NG, Semchishen SN, Geoffroy M, Sikora L, Wafy G, Hsueh L, Khan H, Edwards J, Gravel C, Ferro MA, Colman I (2025). Family dynamics and self-harm and suicidality in children and adolescents: a systematic review and meta-analysis. Lancet Psychiatry.

[64] Bilello D, Townsend E, Broome MR, Armstrong G, Burnett Heyes S (2024). Friendships and peer relationships and self-harm ideation and behaviour among young people: a systematic review and narrative synthesis. Lancet Psychiatry.

