# Supplementary material for: Development and Internal Validation of an Interpretable Machine Learning Model for Identifying Past-Year Nonsuicidal Self-Injury Among Adolescents With Depression: Retrospective Study
Source: JMIR Pediatr Parent. 2026 Oct 8;9:e95340. doi: 10.2196/95340 (PMC13691459; doi:10.2196/95340)
Supplement: Supplementary file 1 [file pediatrics_v9i1e95340_app1.docx]

**Supplementary Table Drafts**

**Supplementary Table S1. Training-set cross-validation and selected hyperparameters**

| Model | Mean CV AUROC (SD) | Search iterations | Selected hyperparameters |
| --- | --- | --- | --- |
| SVM RBF | 0.720 (0.046) | 30 | gamma=0.001; C=10.0 |
| Logistic regression | 0.716 (0.049) | 7 | solver=lbfgs; penalty=l2; C=0.03 |
| SVM linear | 0.716 (0.025) | 6 | C=0.1 |
| Decision tree | 0.709 (0.054) | 30 | min_samples_split=2; min_samples_leaf=20; max_depth=2; criterion=entropy; ccp_alpha=0.005 |
| Random forest | 0.703 (0.050) | 30 | n_estimators=1500; min_samples_split=5; min_samples_leaf=12; max_features=0.5; max_depth=15; criterion=gini; bootstrap=True |
| SVM sigmoid | 0.702 (0.062) | 30 | gamma=0.1; coef0=-1.0; C=0.1 |
| SVM polynomial | 0.680 (0.037) | 30 | gamma=0.01; degree=3; coef0=0.5; C=1.0 |

*Note. CV indicates 5-fold stratified cross-validation conducted exclusively within the training set.*

**Supplementary Table S2. Paired DeLong comparisons between the random forest and alternative models**

| Comparison | RF AUROC | Comparator AUROC | Difference | 95% CI for difference | Unadjusted P | Holm-adjusted P |
| --- | --- | --- | --- | --- | --- | --- |
| RF vs Logistic regression | 0.681 | 0.620 | 0.061 | -0.027 to 0.149 | .175 | .811 |
| RF vs Decision tree | 0.681 | 0.647 | 0.034 | -0.015 to 0.082 | .172 | .811 |
| RF vs SVM linear | 0.681 | 0.613 | 0.068 | -0.034 to 0.170 | .192 | .811 |
| RF vs SVM RBF | 0.681 | 0.619 | 0.062 | -0.027 to 0.152 | .170 | .811 |
| RF vs SVM polynomial | 0.681 | 0.577 | 0.104 | 0.011 to 0.197 | .029 | .171 |
| RF vs SVM sigmoid | 0.681 | 0.617 | 0.064 | -0.026 to 0.153 | .162 | .811 |

*Note. Positive differences favor the random forest.*

**Supplementary Table S3. Sex- and residence-specific performance of the focal random forest**

| Subgroup | n (NSSI/non-NSSI) | AUROC (95% CI) | AUPRC (95% CI) | Accuracy | Sensitivity | Specificity | PPV | Brier score |
| --- | --- | --- | --- | --- | --- | --- | --- | --- |
| Male† | 26 (15/11) | 0.776 (0.576-0.945) | 0.874 (0.761-0.972) | 0.577 | 0.267 | 1.000 | 1.000 | 0.246 |
| Female† | 97 (67/30) | 0.665 (0.548-0.777) | 0.822 (0.752-0.891) | 0.691 | 0.821 | 0.400 | 0.753 | 0.204 |
| Urban† | 74 (50/24) | 0.718 (0.587-0.835) | 0.836 (0.753-0.918) | 0.662 | 0.660 | 0.667 | 0.805 | 0.210 |
| Rural† | 49 (32/17) | 0.619 (0.445-0.778) | 0.768 (0.663-0.873) | 0.673 | 0.812 | 0.412 | 0.722 | 0.217 |

*Note. †Labels assume Gender code 1=male, code 2=female, Residence code 1=urban, and code 2=rural; verify against the source codebook. Subgroup analyses were exploratory.*

**Supplementary Table S4. Comparison of the top 20 SHAP-ranked variables across importance methods**

| Variable | Gini importance | Gini rank | Mean \|SHAP\| | SHAP rank | Permutation importance, mean (SD) | Permutation rank |
| --- | --- | --- | --- | --- | --- | --- |
| SI | 0.1569 | 1 | 0.0971 | 1 | 0.1338 (0.0383) | 1 |
| Gender | 0.0958 | 2 | 0.0541 | 2 | -0.0021 (0.0246) | 64 |
| CR | 0.0320 | 3 | 0.0148 | 3 | 0.0015 (0.0073) | 18 |
| T3 | 0.0301 | 4 | 0.0140 | 4 | -0.0111 (0.0077) | 67 |
| TSH | 0.0282 | 5 | 0.0119 | 5 | -0.0034 (0.0067) | 66 |
| SA | 0.0184 | 10 | 0.0106 | 6 | 0.0052 (0.0047) | 4 |
| CK | 0.0246 | 8 | 0.0104 | 7 | 0.0040 (0.0045) | 6 |
| PRL | 0.0268 | 6 | 0.0103 | 8 | 0.0009 (0.0046) | 29 |
| Age | 0.0166 | 17 | 0.0093 | 9 | 0.0056 (0.0045) | 3 |
| SL | 0.0190 | 9 | 0.0081 | 10 | 0.0006 (0.0060) | 36 |
| PS | 0.0141 | 24 | 0.0079 | 11 | 0.0014 (0.0037) | 19 |
| APOB | 0.0174 | 15 | 0.0066 | 12 | -0.0034 (0.0037) | 65 |
| TG | 0.0152 | 21 | 0.0062 | 13 | 0.0009 (0.0031) | 26 |
| AA | 0.0264 | 7 | 0.0060 | 14 | 0.0136 (0.0052) | 2 |
| ALP | 0.0183 | 11 | 0.0058 | 15 | -0.0003 (0.0040) | 52 |
| IBIL | 0.0163 | 18 | 0.0057 | 16 | 0.0014 (0.0032) | 20 |
| CC3 | 0.0157 | 19 | 0.0054 | 17 | 0.0005 (0.0025) | 37 |
| FBG | 0.0181 | 13 | 0.0053 | 18 | -0.0010 (0.0032) | 60 |
| FA | 0.0153 | 20 | 0.0053 | 19 | 0.0027 (0.0027) | 9 |
| HSCRP | 0.0181 | 12 | 0.0053 | 20 | -0.0008 (0.0030) | 56 |

*Note. Consensus was defined in the analysis code as top-20 by both Gini and SHAP with positive test-set permutation importance. Importance values describe model behavior and do not establish causality.*

Supplementary Figure S1. Held-out test-set AUROCs for random-forest incremental-value and ablation analyses. Error bars represent 95% stratified bootstrap confidence intervals.
